# Meta-omics-aided isolation of an elusive anaerobic arsenic-methylating soil bacterium

**DOI:** 10.1038/s41396-022-01220-z

**Published:** 2022-03-25

**Authors:** Karen Viacava, Jiangtao Qiao, Andrew Janowczyk, Suresh Poudel, Nicolas Jacquemin, Karin Lederballe Meibom, Him K. Shrestha, Matthew C. Reid, Robert L. Hettich, Rizlan Bernier-Latmani

**Affiliations:** 1 Ecole Polytechnique Fédérale de Lausanne (EPFL), Environmental Microbiology Laboratory, CH-1015 Lausanne, Switzerland; 2 Soil Science Group, Institute of Geography, University of Bern, Bern, Switzerland; 3 Bioinformatics Core Facility, Swiss Institute of Bioinformatics, Lausanne, Switzerland; 4 BioSciences Division, Oak Ridge National Laboratory, Oak Ridge, TN USA; 5 Translational Bioinformatics and Statistics, Department of Oncology, Université de Lausanne, Lausanne, Switzerland; 6 Genome Science and Technology Graduate School, University of Tennessee, Knoxville, TN USA; 7 School of Civil and Environmental Engineering, Cornell University, Ithaca, NY USA

**Keywords:** Metagenomics, Biogeochemistry, Soil microbiology, Transcriptomics, Proteomics

## Abstract

Soil microbiomes harbour unparalleled functional and phylogenetic diversity. However, extracting isolates with a targeted function from complex microbiomes is not straightforward, particularly if the associated phenotype does not lend itself to high-throughput screening. Here, we tackle the methylation of arsenic (As) in anoxic soils. As methylation was proposed to be catalysed by sulfate-reducing bacteria. However, to date, there are no available anaerobic isolates capable of As methylation, whether sulfate-reducing or otherwise. The isolation of such a microorganism has been thwarted by the fact that the anaerobic bacteria harbouring a functional arsenite *S*-adenosylmethionine methyltransferase (ArsM) tested to date did not methylate As in pure culture. Additionally, fortuitous As methylation can result from the release of non-specific methyltransferases upon lysis. Thus, we combined metagenomics, metatranscriptomics, and metaproteomics to identify the microorganisms actively methylating As in anoxic soil-derived microbial cultures. Based on the metagenome-assembled genomes of microorganisms expressing ArsM, we isolated *Paraclostridium sp*. strain EML, which was confirmed to actively methylate As anaerobically. This work is an example of the application of meta-omics to the isolation of elusive microorganisms.

## Introduction

Soil microbiomes represent a rich source of novel metabolisms and taxa [1–4]. However, isolating microorganisms from them to study specific functions can be challenging, and even more so in cases for which the phenotype is not identifiable with high-throughput methods [5, 6]. An example of challenging microorganisms to isolate are anaerobic As-methylating strains. Arsenic methylation, catalysed by arsenite (As(III)) *S*-adenosylmethionine methyltransferase (ArsM, in prokaryotes), entails the binding of one to three methyl group(s) to the As atom [7]. At present, there are no available microorganisms capable of anaerobic As methylation. This is because, adding to the constraints associated with maintaining an anoxic environment [8], there is no assay for ArsM activity that can be adapted for high-throughput assessment, despite recent endeavours [9]. Arsenic methylation occurs in anoxic flooded rice paddy soils, is mediated by soil microorganisms [10], and results in the accumulation of methylated As in rice grains [11]. The bioaccumulation of methylated As in rice grains is considerably more efficient than that of inorganic As [12, 13].

The gene encoding ArsM (*arsM*) has been identified in phylogenetically diverse soil microorganisms [14–17]. Anaerobic As methylation is expected to produce a toxic trivalent monomethylated As species (MMAs(III)). The function of this transformation is hypothesised to be microbial warfare, by which the As-methylating organism inhibits microbial competitors via the production of MMAs(III) [18, 19]. If that is confirmed, it is conceivable that As methylation may not occur in pure cultures but only in microbial communities, triggered by metabolites produced by the microbiota. Alternatively, *arsM*-harbouring microorganisms that express As(III) efflux pump(s), the major pathway of As resistance within bacteria [20], may not methylate As due to the efficient removal of As(III) from the cytoplasm, which is the location of ArsM [21, 22]. This effect could be direct, i.e., insufficient substrate concentration, or indirect, i.e., the intracellular As(III) concentration is too low to induce *arsM* expression. Either occurrence (microbial warfare or rapid As(III) efflux) would render the isolation of pure cultures of As-methylating anaerobes very challenging using standard approaches. The latter hypothesis is supported by recent work showing the lack of As methylation by anaerobic pure cultures harbouring functional ArsM enzymes [22].

An additional complexity is evidence for the fortuitous methylation of As upon cell lysis and the release of methyltransferases. This fortuitous activity was suggested for the methanogen *Methanosarcina mazei*, for which As methylation was initiated only when cell viability decreased [22], and by the in vitro methylation of various metals, including As, by MtaA, a methyltransferase involved in methanogenesis [23]. Thus, As methylation activity in cultures incubated beyond the exponential phase may simply be an experimental artefact [22]. Finally, the detection of methylated As requires relatively complex analytical tools (high pressure liquid chromatography coupled to inductively-coupled plasma mass spectrometry, HPLC-ICP-MS) that do not lend themselves readily to high-throughput screening of a large number of colonies [9]. As a result of these challenges, there are no anaerobic microorganisms available known to actively methylate As despite many efforts to identify them. In one instance, researchers had identified a Gram-positive sulfate-reducing bacterium (SRB) [24] that was reported to methylate As but this isolate is no longer available, precluding further investigation.

Thus, this study aimed to conclusively identify an active anaerobic As methylator in soil-derived microbial cultures using a multi-omics approach. The experimental strategy was to build Metagenome-Assembled Genomes (MAGs) from metagenomic data and to identify the subset of MAGs harbouring the gene *arsM* that also expressed the *arsM* RNA transcript (metatranscriptomics) and/or the enzyme ArsM (metaproteomics). Based on the genetic information from the target MAG, an isolation strategy was devised that allowed the recovery of a pure culture, later confirmed to be a novel anoxic As-methylating strain.

## Materials and methods

### Rice paddy soil microbiomes

The soil-derived cultures consisted of two anaerobic microbial enrichments derived from a Vietnamese rice paddy soil and described in Reid et al. [25]. The microbiota from the first soil-derived microbiome was grown in ¼ strength tryptic soy broth (TSB) medium (7.5 g l^−1^ TSB), used previously to enrich As-methylating microbes from a lake sediment [26], and henceforth referred to as the TSB culture. The medium for the second soil-derived microbiome, in addition to ¼ strength TSB, included electron acceptors and two additional carbon sources to simultaneously allow the growth of nitrate-, iron-, and sulfate-reducers, as well as fermenters and methanogens (EA medium: 5 mM NaNO_3_, 5 mM Na_2_SO_4_, 5 mM ferric citrate, 0.2 g l^−1^ yeast extract (Oxoid, Hampshire, UK) and 1 g l^−1^ cellobiose, pH 7). This enrichment will be referred to as the EA culture. Both media were boiled, cooled down under 100% N_2_ gas and 50 ml of medium were dispensed into 100-ml serum bottles. The bottle headspace was flushed with 100% N_2_ gas prior to autoclaving. All culture manipulations were carried out using N_2_-flushed syringes and needles. Cultures were grown at 30 °C. Growth was quantified using optical density at 600 nm (OD_600_).

### Arsenic methylation assays

Pre-cultures from each enrichment were started from −80 °C glycerol stocks. The EA culture started from the glycerol stock was transferred only after a dark precipitate, presumably iron sulfide resulting from sulfate reduction, was formed. The first experimental set-up consisted of bottles containing medium amended with As(III) as NaAsO_2_ (+As condition) pre-inoculation or unamended (no-As control). For this set-up, cell pellets were sampled for DNA sequencing and proteome characterisation during the stationary phase, and for RNA sequencing at the mid-exponential growth phase (see Supplementary Figs. S1, S2 and S3 in Supplementary Information (SI)). In a second experimental set-up, cultures were grown in unamended (no As(III) added) medium and As(III) was added at the mid-exponential growth phase. For this set-up, cell pellets were sampled before (no-As control) and 30 min after As amendment (+As condition) and were used solely for a second transcriptomic analysis. Triplicate biological experiments were performed for each condition (no-As, +As) and per soil-derived enrichment and were used for DNA and RNA sequencing and metaproteome characterisation. Sampling for soluble As species, determination of As speciation, and total As concentration are described in SI.

### DNA sequencing and metagenomic analysis

DNA was extracted from the pellet (10 min, 4500 × *g*) of 4 ml of culture using the DNeasy Power Soil Kit (Qiagen, Hilden, Germany) homogenising with a Precellys 24 Tissue Homogeniser (Bertin Instruments, Montigny-le-Bretonneux, France) (6,500 rpm for 10 s, repeated 3× with 10 s pause intervals). Metagenomic sequencing was performed by the Genomics Platform of the University of Geneva, Switzerland (iGE3) on a HiSeq 4000 (Illumina, San Diego, CA, US). Libraries were multiplexed and prepared using 100-base reads with paired ends according to the Nextera DNA Flex Library Preparation Kit protocol (Illumina). The quality of sequence reads was assessed with FastQC [27] and duplicated reads eliminated by FastUniq [28]. Reads from all biological replicates within the same experimental condition were assembled into contigs using MegaHit [29]. The contig abundance was determined by aligning the sequencing reads from each biological replicate back to the assembled contigs using Kallisto [30, 31]. The abundance for each gene was considered equivalent to the abundance of the contig in which it was encoded. Gene abundance is reported as ‘transcripts per million’ (TPM), referred to as TPM-DNA when used for gene abundance. TPM includes normalisation for gene length and read sequencing depth [32]. Prodigal was used for the prediction of protein-coding genes [33], generating protein sequence libraries for each culture (EA, TSB) and condition (no-As control, +As condition). The annotation server GhostKOALA [34] was used to assign a KEGG Orthology (KO) database number to each protein-coding gene to identify its encoded function and taxonomic category. The 16S small subunit (SSU) rRNA sequences were identified in the contigs and their taxonomy assigned by Metaxa2 [35]. The relative abundance of the 16S SSU rRNA sequences identified in each of the four metagenomes was quantified using the Kallisto-calculated contig abundance. Contigs with length >2000 bp were clustered into bins based on composition and coverage using CONCOCT [36], MetaBAT2 [37] and MaxBin 2.0 [38]. The final bin set was obtained by using the Bin_refinement module from MetaWRAP [39]. Completeness, contamination, strain heterogeneity and community (%) in contigs for each bin were calculated using CheckM [40]. Matching bins between the no−As and +As metagenomes, and between the EA +As and TSB +As metagenomes were identified by pairwise comparison of the predicted genomes using dRep [41]. Bins with an average nucleotide identity >95% were considered identical genomes.

### RNA sequencing and metatranscriptomic analysis

Each culture (5 ml) was harvested at mid-exponential phase for metatranscriptomic analysis. The cells were lysed and the RNA purified using the RNeasy Mini Kit following the manufacturer’s instructions (RNAprotect Bacteria, Qiagen). The purified RNA was DNase-I treated (Promega, Madison, WI, US) (1 h, 37 °C) and cleaned using the RNeasy Mini Kit a second time. Ribosomal RNA (rRNA) depletion (kit QIAseq FastSelect −5S/16S/23S, Qiagen), library preparation using single-end 100 bases reads (TrueSeq Stranded mRNA, Illumina) and RNA sequencing (on a HiSeq 4000) were performed by the iGE3 Platform. Reads were quality-assessed by FastQC, trimmed by Trimmomatic [42], post-sequencing rRNA-depleted by SortMeRNA [43] and aligned to their corresponding protein sequence library by Bowtie2 [44]. The program featureCounts [45] was employed to count the number of RNA reads aligned to the Prodigal-predicted protein-coding genes. The raw counts were used to calculate the TPM, referred as TPM-RNA when employed for transcript abundance. Finally, to assess RNA expression changes in the +As condition relative to the no−As condition, a differential abundance analysis was performed using the DESeq2 package [46] using the protein sequence libraries from the +As condition to align the RNA reads. A gene was considered to have a significant difference in transcription when the absolute log_2_ fold change was ≥1 (i.e., 0.5 ≥ fold change ≥2) and the adjusted *q* value ≤0.05.

### Metaproteome characterisation and metaproteomic analysis

The metaproteome analysis was performed at Oak Ridge National Laboratory (Oak Ridge, TN, US). Biomass pellets from 100 ml of culture were washed with 100 mM NH_4_HCO_3_ buffer (ABC) (pH 8.0), re-suspended in lysis buffer (4% sodium dodecyl sulfate, 100 mM Tris-HCl, pH 8.0) and disrupted by bead-beating. Lysate proteins were reduced with 5 mM dithiothreitol (30 min, 37 °C), alkylated with 15 mM iodoacetamide (30 min in the dark, room temperature) and isolated by a chloroform-methanol extraction. Extracted proteins were solubilized in 4% sodium deoxycholate (SDC) in ABC and the concentration estimated with a Nanodrop (Thermo Fisher Scientific, Waltham, MA, US). Sequencing-grade trypsin (Promega) at a 1:75 enzyme:protein ratio (w/w) was used to digest the proteins and formic acid (1% final concentration) was used to precipitate the SDC and collect tryptic peptides. Aliquots of 12 μg of peptides were analysed by 2D LC-MS/MS consisting of a Vanquish UHPLC connected to a Q Exactive Plus MS (Thermo Fisher Scientific). Spectral data were collected using MudPIT (multidimensional protein identification technology) as described previously [47, 48]. Peptides were separated in three steps (35, 100, and 500 mM ammonium acetate eluent) with organic gradients after each step. Eluted peptides were measured and sequenced by data-dependent acquisition using previously described parameters [49].

Protein databases were created for the +As experimental condition (EA +As and TSB +As) from the corresponding protein sequence libraries generated by Prodigal. The MS/MS spectra raw files were processed in Proteome Discoverer version 2.4 (Thermo Fisher Scientific) with MS Amanda 2.0 [50] and Percolator [51]. Spectral data were searched against the protein database of the corresponding culture (i.e., EA or TSB). The following parameters were used in the search algorithm MS-Amanda 2.0 to derive tryptic peptides: MS1 tolerance = 5 ppm; MS2 tolerance = 0.02 Da; missed cleavages = 2; carbamidomethyl (C, +57.021 Da) as static modification; and oxidation (M, +15.995 Da) as dynamic modifications. The false discovery rate (FDR) threshold was set to 1% for strict FDR and 5% for relaxed FDR at the peptide-spectrum matched (PSM), peptide, and protein levels. FDR-controlled peptides were then quantified according to the chromatographic area-under-the-curve and mapped to their respective proteins. Areas were summed to estimate protein-level abundance.

For differential abundance analysis of proteins, the spectral data from the no−As control, EA no As and TSB no As, were searched against the EA +As and TSB +As protein databases, respectively. All the above-described parameters were maintained. The proteins with at least one peptide detected were exported from Proteome Discoverer. Protein data matrix from EA no As and EA +As were merged and TSB no As and TSB +As were merged. Protein abundance values were log_2_ transformed, LOESS-normalised among biological replicates and mean-centred across all conditions using the software InfernoRDN [52]. Stochastic sampling of the proteins was filtered by removing the proteins without abundance value in at least two of the biological triplicates in at least one condition (no-As control or +As condition). Remaining missing data were imputed by random numbers drawn from a normal distribution (width = 0.3 and downshift = 2.8 using the Perseus software http://www.perseus-framework.org) [53]. The differentially abundant proteins were identified by Student’s *t* test method with adjusted *q* value ≤0.05. Proteins were further filtered using the absolute log_2_ fold change ≥1.

The isolation of the *Paraclostridium sp*. strain EML is described in SI.

## Results

### Arsenic methylation by soil-derived microbiomes

The first experimental set-up yielded samples for the metagenome, metaproteome and one of the metatranscriptomes (labelled metatranscriptome G for ‘growth in the presence of As’) (Supplementary Figs. S1, S2 and S3). The second set-up, assessing the microbiota’s short-term response to As(III), provided sample for the second metatranscriptome (labelled metatranscriptome R for ‘response to arsenic addition’) (Supplementary Figs. S1-A and S2-A). Both EA and TSB cultures exhibited As methylation, reaching an efficiency of As(III) transformation of 27.7% and 19.5%, respectively (Supplementary Figs. S1 and S2).

### Microbiota composition

**Fig. 1:**
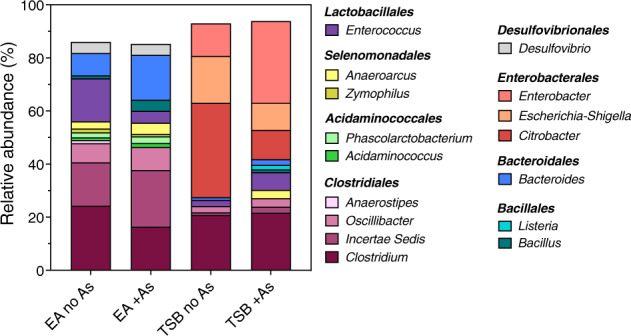
Operational taxonomic units (OTUs) at order and genus level (with >1% relative abundance at genus level) identified from 16S SSU rRNA sequences from soil-derived cultures. Abbreviations: EA no As: EA culture no-As control, EA +As: EA culture +As condition, TSB no As: TSB culture no−As control, TSB +As: TSB culture +As condition. OTUs at the order level are indicated in bold in the legend. Plotted values are the average relative abundance and together with SD values and Student’s *t* test results are available in Supplementary Tables S5 and S6.

The taxonomic classification of 16S SSU rRNA sequences show that, although eukaryotic DNA was also identified, the main fraction of the communities was bacterial (>89.0 ± 0.8% for EA cultures and >98.5 ± 0.3% for TSB cultures, relative abundance) and was distributed amongst eight operational taxonomic units (OTUs) at the order level (Fig. 1 and Supplementary Tables S1–S4). Statistically significant changes (unpaired Student’s *t* test and no significant difference considered when *p* value >0.05) in the OTUs relative abundances, +As condition versus no-As control, are described in SI and summarised in Supplementary Tables S5 and S6.

### MAG selection

The contigs from the four metagenomes, EA (+As, no−As control) and TSB (+As, no−As control), were clustered separately into bins. High-quality (≥90% completeness and ≤5% contamination) bins were designated as MAGs [54]. For the +As condition, the parsing process led to a total of 36 MAGs (Table 1). Additionally, matching bins were sought in the bins from the no-As control cultures (Supplementary Tables S7 and S8). Only one of the 36 MAGs in the +As condition was left unpaired (TSB MAG 8).

**Table 1:** Metagenome-assembled genomes (MAGs) from EA (upper Table A) and TSB (lower Table B) cultures in the +As condition.

A										
MAG	Bin	Marker lineage^a^	Completeness (%)	Contamination (%)	Strain heterogeneity (%)	Genome size (Mbp)	Community (%)	GC content	ArsM-encoding genes	Binner^b^
1	36	*Bacteroidales* (o)	98.5	0.4	0.0	3.8	7.26 ± 0.32	39.1	0	A
2	15	*Clostridiales* (o)	98.7	0.0	0.0	2.2	0.52 ± 0.06	58.4	0	B
3	21	*Clostridiales* (o)	95.2	0.0	0.0	4.3	5.73 ± 0.24	28.5	0	C
4	4	*Clostridiales* (o)	90.7	0.0	0.0	2.1	0.30 ± 0.03	57.5	0	A
5	24	*Clostridiales* (o)	97.8	0.3	0.0	2.0	25.21 ± 0.23	43.2	0	A
6	9	*Clostridiales* (o)	100.0	1.3	50.0	3.2	12.68 ± 0.68	54.9	0	A
7	35	*Clostridiales* (o)	98.0	3.3	55.6	5.3	0.84 ± 0.21	44.0	0	A
8	31	*Clostridiales* (o)	97.9	3.5	0.0	3.8	1.48 ± 0.12	28.2	1	A
9	20	*Clostridium* (g)	97.2	2.2	0.0	3.4	0.70 ± 0.06	30.1	1	C
10	18	*Clostridium* (g)	96.5	2.9	16.7	4.0	0.27 ± 0.05	30.0	0	A
11	11	*Deltaproteobacteria* (o)	99.2	0.7	100.0	3.4	0.63 ± 0.05	57.4	2	BC
12	33	*Deltaproteobacteria* (o)	100.0	1.2	0.0	3.3	12.71 ± 0.49	57.8	3	BC
13	28	*Firmicutes* (p)	99.9	0.0	0.0	2.5	1.02 ± 0.10	47.2	0	B
14	27	*Firmicutes* (p)	91.9	3.3	92.3	3.1	0.84 ± 0.07	49.2	1	BC
15	8	*Lactobacillales* (o)	99.6	0.0	0.0	2.7	3.30 ± 0.61	36.8	0	A
16	1	*Lactobacillales* (o)	99.3	4.6	0.0	4.1	0.99 ± 0.05	39.1	0	BC
17	16	*Selenomonadales* (o)	100.0	1.5	0.0	2.2	2.60 ± 0.24	41.3	0	C
B										
MAG	Bin	Marker lineage^a^	Completeness (%)	Contamination (%)	Strain heterogeneity (%)	Genome size (Mbp)	Community (%)	GC content	N. of ArsM-encoding genes	Binner^b^
1	12	*Clostridiales* (o)	100.0	0.0	0.0	3.1	0.41 ± 0.03	54.8	0	B
2	9	*Clostridiales* (o)	98.9	0.0	0.0	4.7	1.26 ± 0.24	28.4	0	C
3	39	*Clostridiales* (o)	98.0	0.3	0.0	2.0	2.36 ± 0.51	43.2	0	A
4	4	*Clostridiales* (o)	99.3	0.7	100.0	2.7	4.77 ± 3.14	56.1	0	A
5	16	*Clostridiales* (o)	98.7	0.9	0.0	2.8	0.35 ± 0.21	35.7	0	A
6	19	*Clostridiales* (o)	99.2	1.1	0.0	3.5	0.39 ± 0.28	31.2	1	BC
7	1	*Clostridiales* (o)	98.7	1.3	50.0	2.6	0.25 ± 0.07	56.1	1	A
8	15	*Clostridiales* (o)	97.3	2.5	16.7	2.7	0.17 ± 0.02	60.5	0	C
9	28	*Clostridium* (g)	99.3	5.5	23.1	5.6	1.42 ± 0.30	30.1	2	A
10	27	*Clostridium* (g)	98.6	6.9	0.0	4.6	2.10 ± 0.60	32.3	1	A
11	32	*Deltaproteobacteria* (o)	94.8	0.0	0.0	3.1	0.21 ± 0.06	59.3	0	BC
12	38	*Deltaproteobacteria* (o)	98.3	1.8	50.0	3.4	0.81 ± 0.08	57.6	2	BC
13	10	*Enterobacteriaceae* (f)	96.6	0.7	33.3	4.3	0.39 ± 0.08	52.8	0	B
14	42	*Enterobacteriaceae* (f)	95.7	2.1	12.5	5.1	6.77 ± 0.35	56.3	0	BC
15	31	*Firmicutes* (p)	99.9	0.0	0.0	2.4	0.37 ± 0.06	47.6	0	A
16	33	*Firmicutes* (p)	100.0	0.6	0.0	3.2	1.97 ± 1.09	49.1	1	BC
17	7	*Lactobacillales* (o)	99.6	0.0	0.0	2.9	2.64 ± 0.52	36.5	0	C
18	5	*Lactobacillales* (o)	98.9	4.2	0.0	4.1	1.94 ± 0.81	39.1	0	AB
19	36	*Selenomonadales* (o)	100.0	1.5	0.0	2.3	0.81 ± 0.11	41.1	0	A

Marker lineage: taxonomic rank set by CheckM. Completeness and contamination (%): estimated completeness and contamination of genome as determined by CheckM from the presence/absence of single-copy marker genes and the expected colocalization of these genes. Strain heterogeneity: index between 0 and 100 where a value of 0 means no strain heterogeneity, high values suggest the majority of reported contamination is from closely related organisms (i.e., potentially the same species) and low values suggest the majority of contamination is from phylogenetically diverse sources. Proportion of binned proteins assigned to MAG (%): number of protein-coding genes assigned to the MAG divided by the total number of protein-coding genes binned. Community (%): sum of the number of reads mapped to the contigs in each MAG divided by the total number of reads mapped to all contigs including the unbinned contigs, and normalised to MAG size, assuming an average genome size for all unbinned populations.

^a^(p) phylum, (o) order, or (g) genus.

^b^A,B and C refer to MetaBAT 2, MaxBin 2.0 and CONCOCT respectively.

For each MAG, a lineage was assigned by CheckM, based on lineage-specific marker genes [40]. The MAGs identified belonged to the phyla: *Firmicutes* (orders *Clostridiales*, *Selenomonadales* and *Lactobacillales*, and the genus *Clostridium*), *Proteobacteria* (*Enterobacteriaceae* family and *Deltaproteobacteria* class) and *Bacteroidetes* (order *Bacteroidales*). Fifteen MAGs presented non-zero strain heterogeneity (Table 1), an index of the phylogenetic relatedness of binned contigs based on the amino acid identity of the encoded proteins. For ten MAGs, the value is ≥50%, suggesting some phylogenetic relation with the contaminating strains. Five MAGs had heterogeneity values ≤33.33%, suggesting contamination with microorganisms that are not closely related. In the remaining 21 MAGs, the strain heterogeneity is 0%, i.e., no strain heterogeneity or no contamination (Supplementary Tables S7 and S8).

Changes in the relative abundance of MAGs (no-As control vs. +As condition), relatedness of the +As EA and TSB microbial communities, along with the presence, transcription and translation of genes encoding key enzymes from major metabolic pathways of each MAG in the +As condition are included in SI.

### Arsenic resistance genes

**Fig. 2:**
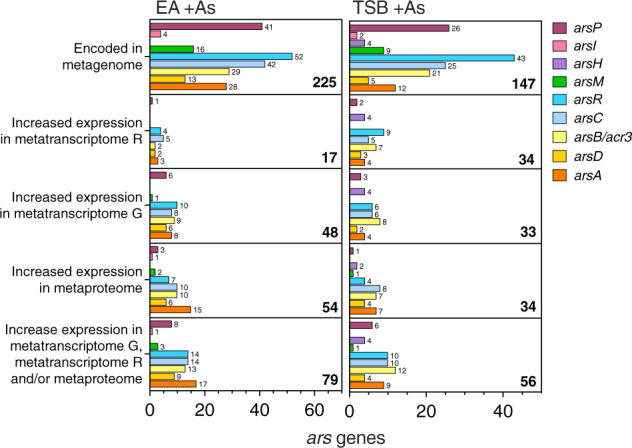
Number of *ars* genes, encoded in the +As condition cultures and with increased expression in metatranscriptomes/metaproteome relative to no−As controls. Number of *ars* genes encoded in metagenomes and with increased expression in metatranscriptomes, R or G, or metaproteomes and the non-redundant overlap between genes with increased expression in metatranscriptomes and/or metaproteomes from +As condition EA (left panels) and TSB (right panels) cultures. Bar length and numbers on the right side of the bars correspond to the number of genes per *ars* gene category. Bold numbers on the lower left corner of each panel correspond to the sum of all *ars* genes per category.

The metagenomic libraries from the +As condition of the EA and TSB cultures were mined for arsenic resistance (*ars*) genes and their encoded proteins (the pipeline is described in SI). A total of 309 and 282 genes were annotated as *ars* genes in the EA and TSB +As metagenomic libraries, respectively (Supplementary Tables S9 and S10). Of those, 255 and 226 were considered correctly annotated as *ars* genes based on BLAST and HMMER (refer to SI for pipeline), and 225 and 147 had above-threshold DNA abundances, respectively (Fig. 2) (refer to SI for abundance threshold values). Individual abundance values of *ars* genes, transcripts and proteins in the +As condition and the no-As control and their transcript and protein relative abundance values in the +As condition vs. the no-As control for each MAG group from the EA and TSB cultures are available in Supplementary Tables S11 and S12, respectively.

The *ars* genes encode proteins involved in the detoxification of As oxyanions: *arsB* and *acr3*, encoding As(III)-efflux systems; *arsA*, encoding the ATPase energising the efflux of As(III) and As(III) chaperone; *arsD*, encoding a weak *ars* operon repressor [55]; *arsC1* and *arsC2*, encoding As(V) reductases coupling As reduction to the oxidation of glutaredoxin or thioredoxin, respectively; and *arsR* genes encoding As(III)-regulated repressors (ArsR1, ArsR2, and ArsR3) classified based on the location of the As(III)-binding cysteine residues [56–58].

The most common *ars* genes in EA and TSB culture metagenomes were *arsR*, *arsC*, and *arsP* (Fig. 2). The first two genes are part of the canonical *ars* operon *arsRBC* [59], whilst *arsP*, encoding a recently discovered membrane transporter, has been found to be widely distributed in bacterial genomes [20]. Most of the surveyed *arsP* genes, 57% in EA and 50% in TSB, are encoded in putative *ars* operons, represented by *ars* genes contiguously encoded in the same contig (Supplementary Tables S11 and S12), supporting their As-related function and correct annotation. The next most abundant genes were those responsible for As(III) efflux (*arsB*, *acr3*, and *arsA*), typically found in organisms living in reducing environments in association with *arsC* [16, 60]. Finally, *arsM* and the two genes, *arsI* and *arsH*, encoding MMAs(III)-resistance mechanisms, were the least recurrent genes in the metagenomes. The results of gene and protein relative expression vs. the no-As control of the *ars* genes involved in the metabolism of inorganic As in the MAGs are described in SI.

### Arsenic-methylating MAGs

**Fig. 3:**
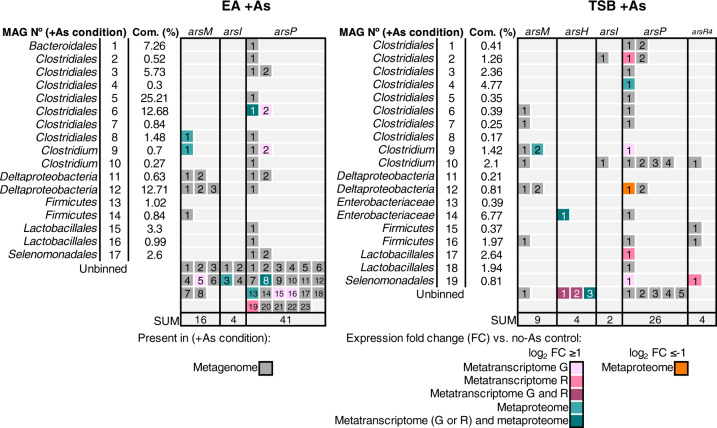
Distribution of *ars* genes involved in methylated arsenic metabolism encoded in MAGs from the +As condition and differentially expressed in metatranscriptomes/metaproteome relative to the no-As EA control. Each numbered box represents an *ars* gene. The number in each box corresponds to the “Numbering” column in Supplementary Tables S11 and S12 where individual gene abundance and fold change values can be found. Com. (%): community (%) as defined in caption from Table 1.

The *arsM* gene can be expressed at similar, or slightly different levels in the absence or presence of As(III) in some organisms [61, 62], but expressed at significantly higher levels in the presence of As(III) in others [63–66]. Thus, we sought to identify *arsM* genes transcribed and ArsM proteins showing increased expression in the +As condition relative to the no−As control (Fig. 3) but also those simply exhibiting expression, not necessarily increased relative to the control (Supplementary Fig. S4).

Sixteen phylogenetically distinct *arsM* genes were identified in the EA +As metagenome, but increased transcriptome reads or peptides (relative to the no-As control) were only detected for three genes (Fig. 3). The first is an *arsM* in *Clostridiales* EA MAG 8 classified by GhostKOALA as belonging to *Paeniclostridium sordellii* (EA MAG 8, *arsM-*1, psor type strain, in Supplementary Table S11). The second was found in *Clostridium* EA MAG 9, also detected in the metaproteome, and the GhostKOALA taxonomic classification of the corresponding gene (EA MAG 9, *arsM-1* in Supplementary Table S11) revealed that it was attributed to the unclassified species *Ruminococcaceae bacterium* CPB6 (Fig. 3, Supplementary Table S11) [67]. Finally, the third *arsM* was obtained from transcriptomic data but not clustered in any EA MAG (EA unbinned, *arsM-5* in Supplementary Table S11) and likewise classified as pertaining to *Paeniclostridium sordellii*.

In the TSB +As metagenome, nine distinct *arsM* genes were identified but none were detected in the metatranscriptome and only one exhibited increased expression in the metaproteome (Fig. 3). It corresponds to an *arsM* gene from MAG 9 (TSB MAG 9, *arsM-2* in Supplementary Table S12). The expressed ArsM protein was assigned by GhostKOALA to a *Clostridiales* strain: *Clostridium botulinum* (*cby* type strain) (TSB MAG 9, *arsM-2*) (Fig. 3, Supplementary Table S12). Finally, there was one *arsM* expressed in the TSB +As metaproteome but with no increased expression relative to the no-As control, it was classified as *Ruminococcaceae bacterium* CPB6 (TSB MAG 9, *arsM-1*) (Supplementary Fig. S4), the same organism identified in the EA culture (EA MAG 9, *arsM-1*).

In addition to evidence for active As methylation, there was evidence for active detoxification of methylated arsenic. Indeed, the metagenome included genes encoding proteins involved in the metabolism of methylated As such as *arsH*, *arsI, arsP*, and *arsR4* (Figs. 2 and 3). These genes encode proteins involved in the detoxification of methylated arsenic like MMAs(III) and roxarsone: the oxidase ArsH, responsible for the oxidation of trivalent methylated As to the less toxic pentavalent form [68]; the demethylase ArsI that removes methyl groups from the As atom [69]; and the transmembrane transporter ArsP, thought to efflux methylated As [70]. The *arsR4* gene encodes an atypical MMAs(III)-responsive ArsR repressor, containing only two conserved cysteine residues [71]. The *Enterobacteriaceae* TSB MAG 14 exhibited activity of the oxygen-dependent ArsH protein [68] (Fig. 3). An *arsR4*, shown to induce expression of *arsP* in the presence of MMAs(III) [71], had increased transcription along with an *arsP* encoded in the same contig in the *Selenomonadales* TSB MAG 19 (Fig. 3, Supplementary Table S12). Both gene transcripts were <5 TPM-RNA (Supplementary Table S12) and thus, were not considered as transcribed in Supplementary Fig. S4. Finally, an ArsI protein, taxonomically related to class *Clostridia* ([*Eubacterium*] *rectale*), was expressed but encoded in an unbinned gene from the EA culture (Fig. 3, Supplementary Table S12).

### Isolation of an arsenic-methylating anaerobic microorganism

Based on the analysis of the active metabolic activity from the EA MAG 8, expressing an ArsM (Supplementary Fig. S5), an appropriate selective medium was identified for its isolation. We utilised the fact that this MAG harbours and expresses the anaerobic assimilatory sulfite reductase encoded by the *asrABC* operon which is responsible for the NADH-dependent reduction of sulfite to sulfide [72–74] in sulfite-reducing *Clostridia* (SRC). From the nine Clostridia MAGs, only two expressed this capability in the EA microbiome (Supplementary Fig. S5). Thus, the isolation relied on growing the EA culture on agar medium selective for the SRC phenotype. In TSC agar, designed for the enumeration of *Clostridium perfringens* in food [75], the colonies from SRC are black, as the ammonium ferric citrate forms iron sulfide during sulfite reduction. Additionally, D-cycloserine acts a selective agent for the isolation of *Clostridia* strains [76] while inhibiting facultative anaerobes [75]. Finally, the bromocresol purple contained in the agar allows the identification of sucrose fermenters, resulting in a change of colour from purple to yellow. As none of the genes involved in sucrose transport or hydrolysis were binned in EA *Clostridiales* MAG 8 (Supplementary Fig. S5), only non-sucrose fermenting black colonies were considered. Those colonies were selected and using a colony PCR screen specifically targeting the *arsM* gene of EA MAG 8, we isolated a *Clostridiales* strain encoding the gene of the expressed ArsM in the EA MAG 8 (protein id k119_30669_28, Supplementary Table S11) (Supplementary Fig. S6).

**Fig. 4:**
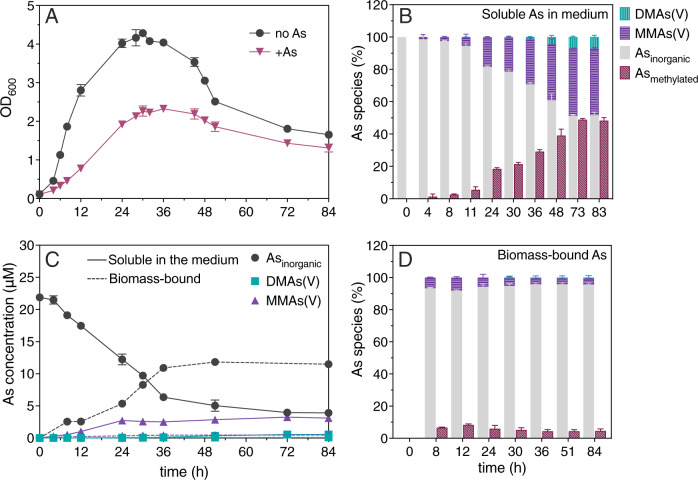
Isolate *Paraclostridium* sp. EML. **A** Growth as OD_600_ with 25 μM As(III) and without, **B** proportion of soluble arsenic species in filtered medium containing 25 μM As(III), **C** concentration of arsenic species soluble in filtered medium containing 25 μM As(III) (solid lines) and biomass-bound (dashed lines) and **D** proportion of biomass-bound arsenic species. Data points and bars represent the mean value and error bars, plus and minus one standard deviation. Individual values for each measurement and biological replicate are available in Supplementary Tables S23 and S24.

The isolate consists of non-sucrose-fermenting, rod-shaped and spore-forming bacteria forming convex and circular black colonies on TSC agar (Supplementary Figs. S7 and S8). The BLAST (NCBI) search of the 16S rRNA sequence gives >99% identity to *Paraclostridium* strains (Supplementary Table S13). On the basis of the 16S rRNA sequence, we assign the following name to the bacterium: “*Paraclostridium* species str. EML”. Strain EML was tested for As methylation under anaerobic conditions with 25 μM As(III). The growth of strain EML was hindered by As(III) (Fig. 4A) and starting from ~4 h, the isolate transformed As(III) to monomethylated soluble As representing 48.3 ± 1.5% of the soluble arsenic in the culture after 83 h (panels B and C from Fig. 4). A fraction (14.7 ± 0.6 μM) of the arsenic was found associated with biomass almost exclusively as inorganic As (Fig. 4D).

## Discussion

Our results demonstrate the successful translation of multi-omic information to a specific strategy for targeted microbial isolation. The metagenomes from the anaerobic soil-derived cultures identified the potential for As methylation in microorganisms from diverse taxa. While there were a large number of *ars* genes in the metagenomes, only a small proportion was transcribed or translated in the presence of As when compared to the no-As control (Fig. 2). This contrast was particularly evident for the gene responsible for As methylation, *arsM*. The post-genomic approaches of community gene and protein expression in TSB as in EA soil-derived microbiomes clearly pointed to the active As-methylating role of various fermenting bacteria from the order *Clostridiales*. This information paved the way for the identification of As-methylating microorganisms and the successful isolation of an anaerobic As methylator.

The TSB and EA media were chosen to selectively enrich for putative As methylators from the microbial soil community based on the study from Bright et al., in which lake sediments enriched in TSB medium, either sulfate-amended or unamended, were shown to have greater As methylation rates than in iron- or manganese-reducing TSB cultures [26]. The selected media caused a great shift in the original soil microbial diversity [25] along with the loss of putative As-methylating microorganisms. Nonetheless, the As-methylating TSB and EA soil-derived cultures offered the opportunity to study active As methylation from paddy-soil microbiota in an environment that is less complex than soil but that remains environmentally relevant. In contrast to soil slurries, the absence of soil minerals in the soil-derived cultures facilitated the detection of soluble methylarsenicals and the extraction of DNA, RNA and proteins. The multi-omic approach made it possible to identify putative microorganisms driving As methylation and their metabolism. Targeting a specific *arsM* gene rather than the synthesis of methylarsenicals greatly accelerated colony screening, as colony PCR could be employed instead of analytical detection by HPLC-ICP-MS.

**Fig. 5:**
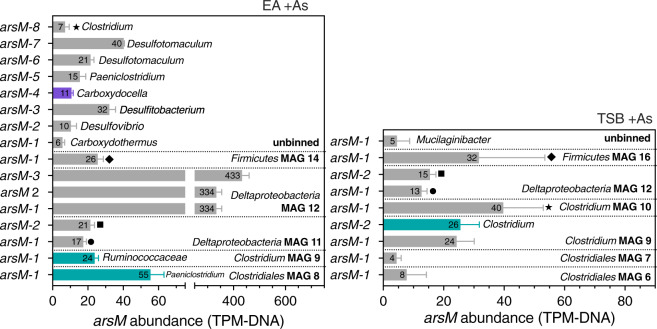
Gene abundance of *arsM* genes in MAGs from the +As condition cultures. Coloured bars correspond to *arsM* genes with increased expression in the metaproteome (blue-green) or in the metatranscriptome G (purple) from +As condition relative to the no-As control in EA (left panel) and TSB (right panel) cultures. The taxonomic classification shown on the right side of the error bars for selected *arsM* genes corresponds to the individual gene classification assigned by GhostKOALA - “Genus” column in Supplementary Tables S11 and S12. Columns with matching symbols on the right side of the error bars, correspond to matching *arsM* genes between the cultures. Individual gene abundance can be found in Supplementary Tables S11 and S12. Numbers inside bar and bar length represent mean and error bars one standard deviation.

Had only the metagenomic approach been implemented, the data would have pointed to SRB MAGs as putative As methylators, as they harboured the most abundant *arsM* genes (Fig. 5). Indeed, SRB have been proposed as drivers of As methylation in rice paddy soils based on the correlation in the abundance of *arsM* and dissimilatory sulfite reductase (*dsr*) genes [77] and RNA transcripts [78], and a decrease in As methylation by the addition chemical inhibitors of dissimilatory sulfate reduction (DSR) [77, 78]. Additionally, the use of degenerate primers for *arsM* amplification may underestimate *arsM* phylogenetic diversity, a drawback overcome by metagenomic and metatranscriptomic sequencing. In the present findings, the SRB *Deltaproteobacteria* MAGs, although actively reducing sulfate (Supplementary Figs. S5 and S9), did not exhibit As-methylating activity as their *arsM* genes were neither transcribed nor translated (Fig. 5). *Desulfovibrio* MAGs were metabolically active in both cultures, but amongst all their encoded *ars* genes, only an *arsR3* exhibited increased expression in the presence of As(III), providing strong evidence for their lack of involvement in As methylation in the TSB and EA cultures.

Previous work had identified another As-methylating *Clostridiales* strain, *Clostridium* sp. BXM [24], that performed fermentation and DSR but that is no longer available. The sole attribution of As-methylating activity to fermenting Firmicutes in that work, along with the isolation of the present sulfite-reducing fermenter, point to a key role for fermenting *Clostridiales* microorganisms harbouring sulfur-related metabolism in As methylation. Other studies have reported an increase in As methylation efficiency after the amendment of sulfate [79] or organic matter to soil [15, 80–82], or after the increase in dissolved organic carbon in soil [83]. The positive impact of sulfate amendment on As methylation was interpreted as pointing to the role of SRB in As methylation [79]. Here, we offer an alternative explanation, supported by examples of organic amendments enhancing As methylation [15, 80–82]. The sulfate amendment could have indirectly increased the availability of short-chain fatty acids through DSR, providing fermentable substrates. Thus, we propose that direct or indirect organic amendments result in the enrichment of fermenting communities, and consequently, in an increase in As methylation.

It was previously proposed that the As-methylating activity of anaerobic microorganisms may be limited by efficient efflux of intracellular As(III) [22], or that it may function as a defensive response against nutrient competition [18]. Indeed, the identification of MAGs exhibiting a detoxification response to methylarsenicals supports the hypothesis of the role of monomethylated As as an arsenic-bearing antibiotic. Although the expression of ArsI and ArsH, catalysing oxygen-dependent MMAs(III)-resistance mechanisms (Fig. 3), is difficult to reconcile with anoxic conditions, it is conceivable that these proteins are capable of additional functions in the absence of O_2_. Up until now, the lack of available anaerobic microbial isolates able to methylate As in vitro [22] precluded the investigation of the hypotheses raised above. This work represents the first study applying a combination of three meta-omic techniques in order to characterise As metabolism in microbial communities and to perform meta-omics-aided isolation of a microorganism [84, 85]. The successful isolation of *Paraclostridium* sp. EML is part of the “new era of omics information-guided microbial cultivation technology” described by Gutleben et al. [84] and represents a milestone to obtain novel targeted microbial isolates from the environment and to elucidate the controls on anaerobic As methylation.

Further work is needed to elucidate why ArsM expression was restricted to members of *Clostridiales* fermenters and did not occur in other organisms harbouring *arsM* genes. The availability of As-methylating anaerobes will allow investigation of why the *arsM* gene evolved under an anoxic atmosphere [86], of the controls on the production of toxic methylated As species in flooded rice paddies, and the development of microbially-mediated remediation technologies for As-contaminated soils via the synthesis of volatile methylarsenicals [87, 88].

## Supplementary information

Supplementary Information (SI)

Supplementary Information (SI) Tables for the research article: Meta-omics-aided isolation of an elusive anaerobic arsenic-methylating soil bacterium

## Data Availability

Metagenomic and metatranscriptomic raw sequencing reads are available at the National Centre for Biotechnology Information (NCBI) Sequence Read Archive (SRA), BioProject PRJNA714492. Data from the meta-omic analyses and source data from figures are available in Zenodo data repository (10.5281/zenodo.4605527).
