# Supplementary material for: Meta-omics-aided isolation of an elusive anaerobic arsenic-methylating soil bacterium
Source: ISME J. 2022 Mar 25;16(7):1740–9. doi: 10.1038/s41396-022-01220-z (PMC9213503; doi:10.1038/s41396-022-01220-z)
Supplement: Supplementary file 1 — Supplementary Information (SI) [file 41396_2022_1220_MOESM1_ESM.pdf]

Supplementary Information (SI) for the research article: Meta-omics-aided isolation of an elusive anaerobic arsenic-methylating soil bacterium

Karen Viacava[1,2], Jiangtao Qiao[1], Andrew Janowczyk[3], Suresh Poudel[4], Nicolas Jacquemin[1,5], Karin Lederballe Meibom[1], Him K. Shrestha[4,6], Matthew C. Reid[7], Robert L. Hettich[4], Rizlan Bernier-Latmani[1*].

[1] Ecole Polytechnique Fédérale de Lausanne (EPFL), Environmental Microbiology Laboratory, CH-1015 Lausanne, Switzerland.

[2] Soil Science Group, Institute of Geography, University of Bern, Bern, Switzerland.

[3] Bioinformatics Core Facility, Swiss Institute of Bioinformatics, Lausanne, Switzerland.

[4] BioSciences Division, Oak Ridge National Laboratory, Oak Ridge, TN, United States.

[5] Translational Bioinformatics and Statistics, Department of Oncology, Université de Lausanne, Lausanne, Switzerland.

[6] Genome Science and Technology Graduate School, University of Tennessee, Knoxville, TN, United States.

[7] School of Civil and Environmental Engineering, Cornell University, Ithaca, NY, United States.

*Corresponding author. E-mail address rizlan.bernier-latmani@epfl.ch. Postal address EPFL ENAC IIE EML CH A1 375 (Bâtiment CH) Station 6 CH-1015 Lausanne Switzerland. Tel. +41 21 69 35001.

SI Materials and Methods

Total arsenic and arsenic speciation analysis

Arsenic speciation was obtained using an HPLC 1260 Infinity or an ICP-QQQ 8900, respective instrument settings in Table S14, coupled to an HPLC 1260 Infinity II (Agilent Technologies, CA, US) with an anion exchange PRP X-100 HPLC column (150 × 4.1 mm I.D., 10 µm, Hamilton, NV, US) (pump: 1ml min^{-1}, injection volume: 15 µl, autosampler: 4°C and column compartment: 20 °C). The eluent was 6.66 mM NH_4NO_3 and 6.66 mM $(NH_4)_2HPO_4$ (pH 6.2 adjusted with HNO_3). The As standards consisted of TMAsO as trimethyl arsine oxide (Argus Chemicals Srl., Italy), DMAs(V) as sodium dimethylarsinate (ABCR, Germany), MMAs(V) as monomethylarsonic acid (Chemservice, PA, USA) and As(V) as $Na_2HAsO_4{\cdot}7H_2O$ (Sigma-Aldrich, MO, USA). The samples were oxidized prior to analysis, due to the co-elution of As(III) and TMAsO species. The remaining As(III) was quantified in its oxidized form as As(V). In this way, As speciation analysis allowed the discrimination of inorganic As (detected as As(V): sum of remaining As(III) plus As(V) produced during incubations) from tri-, di- or monomethylated arsenicals (as TMAsO, DMAs(V) or MMAs(V)) but not the identification of the original redox state of the methylarsenicals as produced by the strains. Total As concentrations were measured using the same ICP-MS instruments in stand-alone mode.

Isolation of Paraclostridium *sp. EML, an anaerobic As-methylating microorganism*

The isolation of the anaerobic *arsM*-expressing microorganism was conducted by using serial dilution agar plate method in an anaerobic chamber (Coy Laboratories, Grass Lake, MI, USA) containing 90% N_2:10% H_2 with less than 5 ppm of O_2. Briefly, 1 ml of cell suspension of the EA culture was serially diluted in a 10-fold series (10^{-1} to 10^{-5} dilutions) using sterile Reinforced Clostridial Broth (RCB) (Oxoid Ltd., Basingstoke, UK). Consecutively, 100 µl of EA cell suspension from each dilution was spread uniformly over the surface of Reinforced Clostridial agar (RCA) (Oxoid Ltd., Basingstoke, UK). The inoculated RCA plates were incubated at 30°C for 24 hours. The single colonies were transferred with sterile toothpicks to tryptose-sulphite-cycloserine (TSC) agar (Merck, Darmstadt), containing 5 g l^{-1} sucrose, 0.04 g l^{-1} bromocresol

purple (Sigma-Aldrich), and 0.4 g l^{-1} de-hydrated D-cycloserine (Sigma-Aldrich), and grown at 30°C for 24 hours.

Colony PCR was performed on black colonies picked from purple TSC agar using the designed specific primers arsM-9F: 5'-TCTAATCTAAGTTGTTGTGGGGAAG-3' and arsM-9R: 5'-TGATATAGATAACCTACCTCCGCC-3', generating a 500 bp amplicon of the *arsM* gene from MAG 8 from the EA culture (Table 1-A) (protein id k119_30669_28, Table S11). Before direct colony PCR, the black colonies were first picked, diluted into 15 μl lysis buffer (0.1% triton X-100 + TE buffer) and boiled at 95°C for 10 min, to release DNA, and then centrifuged (13,000 g, 10 min) to spin down cell debris. The supernatant of the lysate was used as the DNA template for colony PCR.

Each colony PCR consisted of a 25-μl reaction containing: 12.5 μl 2x GoTaq Green Master mix (Promega, UK), 0.5 μl of the reverse and forward primers (10 μM each), 1.5 μl of lysate supernatant as the DNA template, 0.25 μl of 20 mg ml^{-1} bovine serum albumin (BSA) (Sigma-Aldrich), and 10.75 μl sterile DNase RNase Free water. The thermocycling program consisted of an initial denaturation at 95°C for 5 min, 30 cycles of denaturation at 95°C for 40s, annealing at 53°C for 40 sec, and extension at 72°C for 40s, and a final extension at 72°C for 10 min. After purification of the PCR product with Wizard SV Gel and PCR Clean-Up System (Promega, UK), the *arsM* amplicon was sequenced at Microsynth (Balgach, Switzerland). The amplification of full-length 16S rRNA gene was performed using the primers 27F [1] and 1542R [2].

Arsenic methylation assay for Paraclostridium *sp. EML*

The As methylation assay consisted of a time-course experiment. RCB was brought to a boil, then cooled down to room temperature under a gas flow (100% N_2), and dispensed into 200-ml serum bottles (100 ml of medium per bottle) under the same gas atmosphere. The medium was amended with 25 μM As(III) as $NaAsO_2$, and a no-As(III) control was included (three replicates per condition). Bottles were inoculated with 1% inoculum (v/v) of a pre-grown

exponential phase culture. Samples were obtained for soluble and volatile As as well as for microbial growth as previously described for anaerobic cultures [3]. Growth was quantified using OD_{600}. Arsenic bound to the biomass was assessed, as described in [4], by collecting a pellet of 1 ml of culture (13,000 g, 10 min), resuspending in 1 ml lysis buffer (0.1% triton X-100, 0.1% SDS, 10 mM EDTA, and 1 mM Tris−HCl), boiling 95 °C for 18 min with vortexing every 3 min and diluting 10x in 1%HNO_3 prior to analysis for total As and As species.

Arsenic speciation was determined in the step-gradient elution mode with an As Spec anion exchange fast column (50 mm x 4.0 mm, PrinCen, Guangzhou, China) (pump: 1.2 ml min^{-1}, injection volume: 10 μl, autosampler: 4°C and column compartment: 20°C). The mobile phase consisted of eluent A (0.23 ml 69% HNO_3 + 1.8 ml 28% ammonium) and eluent B (2.32 ml 69% HNO_3 + 4.6 ml 28% ammonium). The gradient program was set for 0-80 s 100% eluent A, 81-260 s 100% eluent B, 261-300 s 100% eluent A. Five arsenic standards were prepared, including TMAsO as trimethyl arsine oxide (Argus Chemicals Srl., Italy), DMAs(V) as sodium dimethylarsinate (ABCR, Germany), MMAs(V) as monomethylarsonic acid (Chemservice, PA, USA), and As(V) as $Na_2HAsO_4{\cdot}7H_2O$ (Sigma-Aldrich, MO, USA). Total As concentrations were measured using the same ICP-MS instruments in stand-alone mode. Instrument settings in Table S14.

SI Results

Arsenic methylation by soil-derived microbiomes

The added initial As(III) concentrations, or those after spiking in the second set-up, were 28.3± 2.7 μM and 24.4± 5.8 μM for the EA and TSB experiments, respectively (Figures S1-B and S2-B). A decrease in the total initial soluble As was observed in most cases and could be due either to volatilization or intracellular accumulation of As (not assessed). Soluble methylarsenicals were found in all experiments, mainly mono- and dimethylated As (panels C and D from Figures S1 and S2). Arsenic species were oxidized prior to analysis [3]. Thus, As-speciation analysis discriminated between inorganic As and mono-, di-, or trimethylated arsenicals but did not allow the identification of the redox state of the methylarsenicals. Therefore, even when DMAs(V) and MMAs(V) were measured, they could have corresponded to trivalent species prior to oxidation.

The highest methylation efficiencies were achieved 45 h after inoculation: 27.7% of the initial As(III) for the EA culture, and 19.5% for the TSB culture. Previously, the EA culture was reported to convert 63% of the initial iAs 100 h after inoculation [4]. The variation in methylation efficiency between the previous work is likely due to the difference in sampling time. In the case of TSB experiment, the higher TSB methylation efficiency observed in the metagenome culture may be due to the lower initial concentration (15.1± 2.7 μM), as lower As concentrations have been observed to lead to higher As methylation efficiencies [3].

Abundance changes in microbiota composition

In the no-As control EA culture, *Firmicutes* (76.0 ±0.4%) was the most abundant phylum, particularly members of the order *Clostridiales* (49.6 ±1.6%) and Lactobacillales (16.9 ±1.2%) (Table S5). When the community was grown in the presence of arsenic, the taxa belonging to *Clostridiales* and *Desulfovibrionales* did not present statistically significant changes in abundance (Table S5). In contrast, significant changes were observed in the abundance of taxa from the order *Lactobacillales*, which decreased by two thirds, from 16.9 ±1.2% (no-As

control) to 5.0 ±0.6% (+As condition), from the order *Bacteroidales*, which doubled going from 8.4 ±0.5% to 16.9 ±0.4%. Additionally, *Selenomonadales* and *Acidaminococcales* exhibited a slight but statistically significant increase from 4.2 ±0.5% to 5.1 ±0.3% and from 2.9 ±0.0% to 4.0 ±0.5%, respectively.

The order *Enterobacterales* dominated the microbiota in the TSB no-As control representing 65.5 ±6.4% of the community, followed by orders from the phylum Firmicutes: *Clostridiales* (24.7 ±4.7%), *Lactobacillales* (2.3 ±0.1%) and *Bacillales* (1.1 ±0.4%) (Table S5). The TSB community was altered upon exposure to As and exhibited a statistically significant increase in the order *Lactobacillales* (to 6.8 ±1.96%) and *Bacteroidales* (to 2.33 ±0.57%).

At the genus level (Figure 1, Table S6), the order *Clostridiales* included primarily contributions from members of the genera *Clostridium* and *Oscillibacter* for both EA and TSB. In addition, there was a contribution from organisms for which there is no attribution at the genus level (*Incertae Sedis*) which was especially large in the EA culture. In both cultures, *Bacteroidales* consisted entirely of the *Bacteroides* genus and *Lactobacillales* of the *Enterococcus* genus. Additionally, for EA, the orders *Desulfovibrionales* and *Bacillales* each comprised one genus, *Desulfovibrio* and *Bacillus*, respectively. In the TSB culture, the most abundant genera were *Citrobacter* and *Enterobacter*, in the absence and presence of arsenic respectively, both from the dominant *Enterobacterales* order.

Relatedness of the +As EA and TSB microbial communities

To evaluate the relatedness of the EA and TSB microbial communities, matching bins between the EA and TSB +As condition MAGs were found by pairwise comparison of the predicted genomes. Of the 36 MAGs identified across EA and TSB cultures, 18 (corresponding to 9 pairs) exhibited similarities >98.47% (Table S15), including a *Deltaproteobacteria*, three *Clostridiales*, two *Lactobacillales*, a *Selenomonadales*, and two *Firmicutes*. Thus, approximately half of the MAGs of the EA culture are also present in the TSB culture and vice-versa.

Abundance changes in MAGs

The relative abundance of each MAG in the community was extracted using the *Profile* command in CheckM and reported as community (%). This proportion is calculated as the ratio of the number of reads mapped to the contigs in each MAG and the total number of reads mapped to all contigs (including the unbinned contigs), and adjusted for the size of the MAG (assuming an average genome size for the unbinned fraction). Based on community (%), approximately 77.6% and 38.0% of the EA and TSB+As microbiota, respectively, are represented in the retained MAGs (Table 1). The most abundant MAGs, >5% relative abundance, in the EA community are: *Clostridiales* MAG 5 (25.21 ±0.23%), *Deltaproteobacteria* MAG 12 (12.71 ±0.49%), *Clostridiales* MAG 6 (12.68 ±0.68%), *Bacteroidales* MAG 1 (7.26 ±0.32%) and *Clostridiales* MAG 3 (5.73 ±0.24%); and, in the TSB community is *Enterobacteriaceae* MAG 14 (6.77 ±0.35%).

The changes in community (%) between the +As condition and the no-As control were significant in 14 of 17 the EA MAGs for which a matching MAG was found (Figure S10, upper panel). Some of the changes in the EA MAG abundances agree with the ones observed at the order level in the 16SS rRNA OTUs, e.g., the *Bacteroidales* MAG 1, and *Selenomonadales* MAG 17 increased as did their corresponding orders (Figure 1 and Table S5). However, the increment in both *Deltaproteobacteria* EA MAGs was not reflected in the 16SS rRNA OTUs.

Contrary to EA, most of TSB MAGs did not present statistically-significant abundance changes (Figure S10, lower panel) between the no-As control and +As condition. However, as in the case of EA, some of the changes observed agree with the 16SS rRNA OTU changes like the significant decrease in the *Enterobacteriaceae* MAG 14 as in the *Enterobacterales* order (Figure 1 and Table S5). Finally, most of the TSB MAGs with an equivalent MAG pair in the EA MAGs (Table S15), presented the same increasing or decreasing behaviour in their abundance, except from the *Selenomonadales* MAG that increased its community abundance in the EA +As culture compared to the no-As control while decreasing its abundance in the TSB +As culture compared to the no-As control.

Active metabolic pathways in MAGs

The presence, transcription and translation of genes encoding key enzymes from various metabolic pathways were assessed for each EA and TSB MAG (Figures S5 and S9). As described in the manuscript, a gene was considered as present in the MAG if DNA reads represented >5 TPM-DNA, as transcribed if RNA reads were >5 TPM-RNA and as translated if protein abundance could be calculated from the detected peptides, in at least two of the three biological replicates for each case. Individual values of gene, transcript and protein abundance levels for each MAG, biological replicate and enzyme are available in Tables S16 and S17.

The Deltaproteobacteria MAGs expressed the pathway for dissimilatory sulphate reduction (DSR) in both cultures; the dissimilatory reduction of nitrate to ammonia (DNRA) in the EA culture, presumably due to the presence of nitrate in the medium; and enzymes for acetate (AckA), ethanol (YiaY) and butanediol fermentation (as indicated by the expression of the acetolactate synthase, synthesizing a butanediol intermediary).

All members from the *Enterobactericeae* family are able to couple nitrate reduction to glucose fermentation[5]. The *Enterobactericeae* MAGs from TSB, expressed the enzymes needed for denitrification and mixed-acid fermentation: formate (PfD), acetate (AckA), ethanol (ADH5, and ADHE) and butanediol. *Lactobacillales* MAGs in both enrichments presented the same fermentative metabolisms as *Enterobacterales* plus lactate (LDH) and, in one of them, the amino-acid fermentation (Stickland fermentation).

Finally, in both cultures, the MAGs belonging to the phylum Firmicutes (*Clostridiales*, *Clostridium*, *Lactobacillales*, *Selenomonadales* and *Firmicutes* MAGs) displayed various mixed acid fermentations, acetone-butanol-ethanol (ABE) fermentation and amino-acid fermentation. Key enzymes from the acetyl-CoA (Wood-Ljungdahl) pathway, specially the formate-tetrahydrofolate (THF) ligase (Fhs), were expressed in all of the MAGs belonging to the phylum Firmicutes with the exception of *Selenomonadales* MAGs. Given the highly nutritious broth used for the growth of the communities, i.e., high carbon availability, the transcription and translation of *fhs* could be an indication of an active reverse Wood-Ljungdahl

pathway. This reversal of the Wood-Ljungdahl pathway consists in the oxidation of acetate to hydrogen and carbon dioxide and has been shown to occur in anaerobic environments [6].

Metatranscriptomics and metaproteomics differential analyses in metagenomic libraries

Differential analysis of the metatranscriptomes shows that the presence of As impacted the transcription of a higher proportion of genes in the TSB culture than in the EA culture (Figure S11 and Table S18). For metatranscriptomes R, while in TSB culture the proportion of genes with increased or decreased transcription was almost the same, for EA culture the proportion of genes with increased transcription was greater that of the genes with decrease transcription. Conversely, in metatranscriptome G the proportion of genes with increased transcription was half of the genes with decreased transcription for TSB culture but remained almost the same for EA culture. In the case of the metaproteomes, the proportion of genes with increased expression was greater than the genes with decreased expression for EA and TSB cultures (Figure S12). The genes with differentially abundant RNA transcripts and proteins are enlisted in Tables S19 and S20.

*Identification of arsenic resistance (*ars*) genes*

In addition to GhostKOALA, the protein sequence libraries from EA and TSB of the +As condition were further annotated using the EggNOG server [7] in order to assign a KO number and an orthologous group (OG) to each protein-coding gene. Using the annotation from both servers, the libraries were queried for arsenic resistance (*ars*) genes (Table S21). To verify the annotation of the putative *ars* genes, the amino acid sequences of the encoded proteins were aligned, using BLAST, against representative arsenic-resistance proteins (Table S22) selected based on previous studies [8, 9]. Alignments were considered accurate only when the E-value <0.01. The BLAST annotation was assigned according to the annotation of either the top or the top three reference protein alignments. Further verification of the annotation was done by searching the amino acid sequences against Hidden Markov Model (HMM) profiles from the reference sequences using *hmmscan* in HMMER v3.1b2 package [10] with an E-

value <0.01. One gene annotated by the databases as *arsB,* from EA +As library, was reassigned to *acr3* based on BLAST and HMMER results (protein id k119_31951_258, Table S9).

Increased expression of ars *genes in the +As condition relative to the no-As control*

A gene was considered to be present in the culture if DNA reads represented >5 TPM-DNA (‘transcripts per million’ (TPM), referred to as TPM-DNA when used for gene abundance), considered to be transcribed if >5 TPM-RNA (referred as TPM-RNA when employed for transcript abundance) were detected, and as translated if protein abundance could be calculated from the detected peptides in at least two of the three biological replicates. Additionally, increased expression in the RNA and protein in the +As condition relative to the no-As condition, was considered when the absolute $\log_2$ fold change was ≥1 (i.e., 0.5 ≥ fold change ≥2) and the adjusted *q* value ≤0.05 (Figure 2).

In 22 of the 36 MAGs from the +As cultures, at least one *ars* gene exhibited increased transcription or protein expression relative to the no-As control (Figures S13 and S14). Only four *ars* genes, two *arsR2* in EA, an *arsC1* and an *arsP* in TSB showed a decrease, all four in the metaproteome. With the exception of *Clostridiales* MAG 8 in TSB, all MAGs contained at least one *ars* gene (Figures S13 and S14).

A similar number of *ars* genes had increased transcription, relative to the no-As control, in both metatranscriptomes (R and G) for the TSB +As culture. In contrast, for the EA +As culture, threefold as many genes had increased RNA reads, relative to the no-As control, in metatranscriptome G as compared to metatranscriptome R (bold numbers in Figure 2). There was a significant overlap of *ars* genes, 44% in EA and 64% in TSB, that were detected as transcripts and proteins (Figure S15).

The expression as mRNA transcripts and/or proteins of *arsA*, *arsD*, *arsC*, *acr3*, and *arsB* in 18 of the 22 MAGs with expressed *ars* genes, underscores the predominance of intracellular As(III) efflux as a detoxification strategy in the microbiomes. The vast majority of As(III) efflux genes correspond to *acr3*, the most common membrane-transporter-encoding gene in *ars*

operons [11]. The *arsB* gene was only expressed in the *Enterobacteriaceae* MAGs (TSB MAGs 13 and 14) in TSB (Figure S14). The genes reported as unbinned consist of *ars* genes in contigs that could not be clustered in a MAG or that belonged to unretained, no high-quality bins.

SI Figures

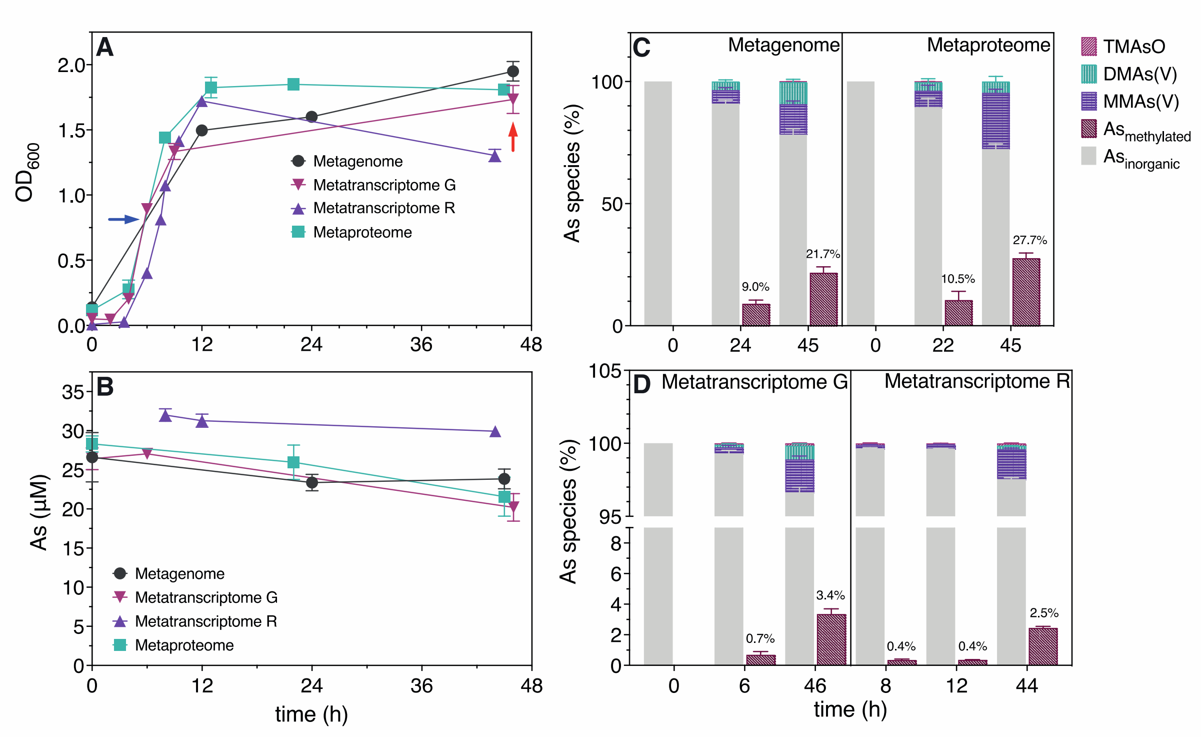

Figure S1. Arsenic methylation in the +As condition EA culture. A) Growth curves from experiments as OD_{600}. Red arrow indicates the samples used for metagenome and metaproteome analyses. Blue arrow indicates the samples used for metatranscriptomes G and R. B) Total soluble arsenic in medium. C) Proportion of arsenic species soluble in medium for metagenome and metaproteome experiments. D) Proportion of arsenic species soluble in medium for metatranscriptome G and metatranscriptome R experiments. OD_{600} corresponds to optical density at 600 nm. Points and bar heights represent mean and error bars plus, minus one standard deviation. Individual values for each measurement and biological replicate are available in Tables S25 and S26.

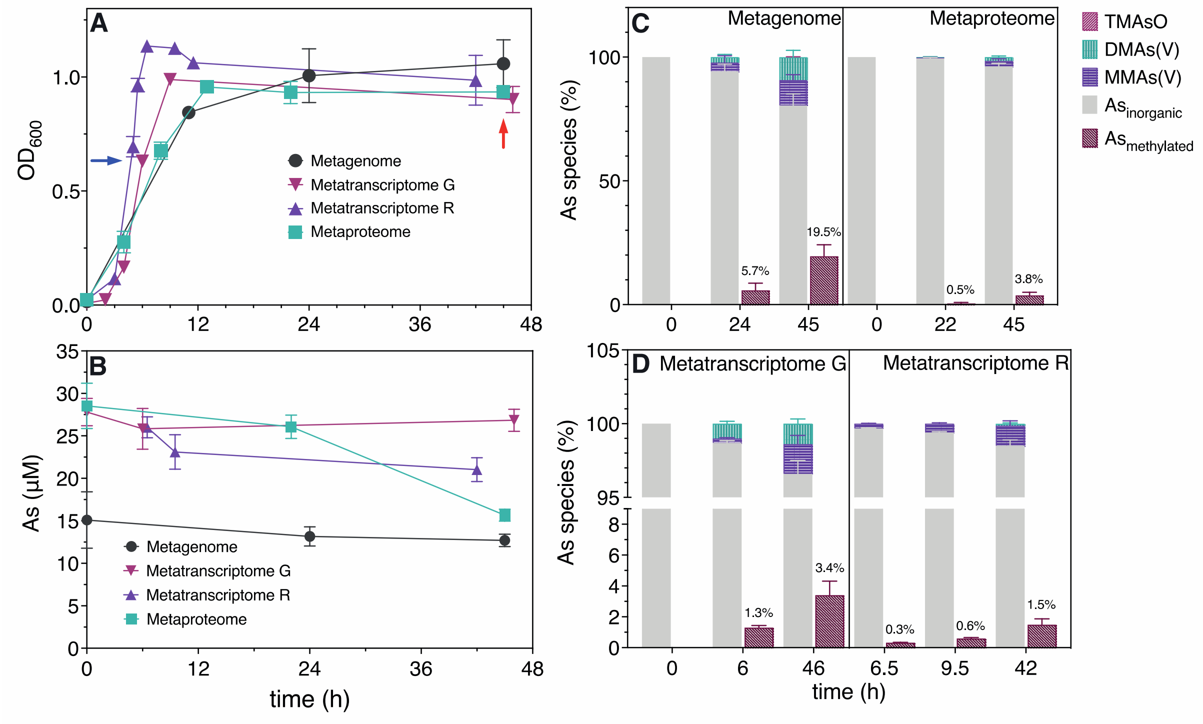

Figure S2. Arsenic methylation in the +As condition TSB culture. A) Growth curves from experiments as OD_{600}. Red arrow indicates the samples used for metagenome and metaproteome analyses. Blue arrow indicates the samples used for metatranscriptomes G and R. B) Total soluble arsenic in medium. C) Proportion of arsenic species soluble in medium for metagenome and metaproteome experiments. D) Proportion of arsenic species soluble in medium for metatranscriptome G and metatranscriptome R experiments. OD_{600} corresponds to optical density at 600 nm. Points and bar heights represent mean and error bars plus, minus one standard deviation. Individual values for each measurement and biological replicate are available in Tables S25 and S26.

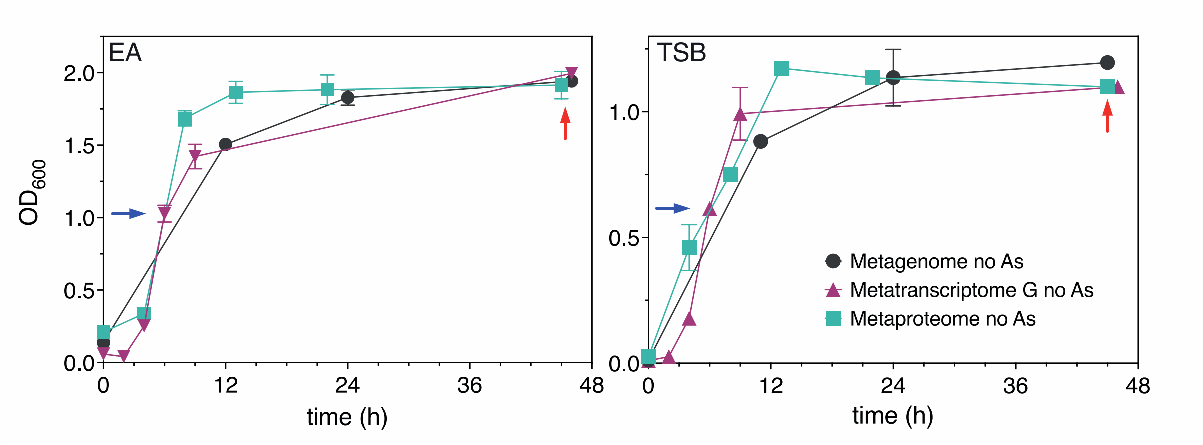

Figure S3. Growth in no-As controls. Growth curves as OD_{600} from no-As controls in EA (left panel) and TSB (right panel) cultures. Red arrow indicates the samples used for metagenome and metaproteome analyses. Blue arrow indicates the samples used for metatranscriptome G. Points represent mean and error bars plus, minus one standard deviation. Individual values for biological replicate are available in Table S25.

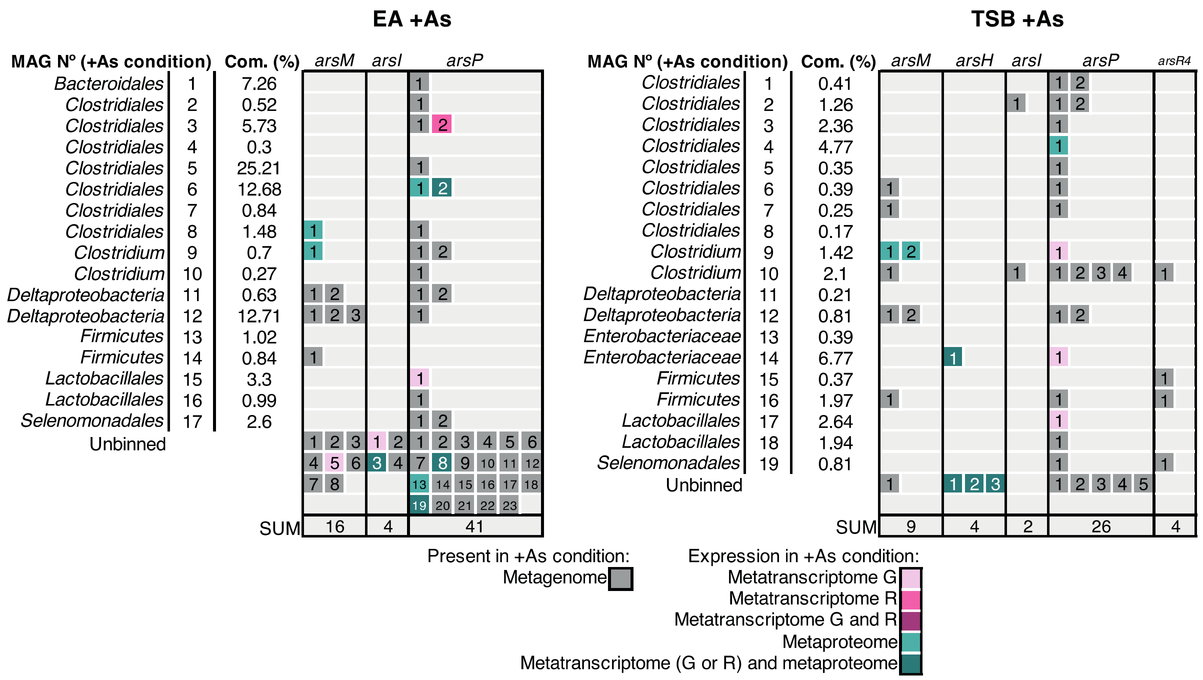

Figure S4. Distribution of *ars* genes involved in methylated arsenic metabolism encoded in MAGs from the +As condition and expressed in metatranscriptomes/metaproteome. Each numbered box represents an *ars* gene. The number in each box corresponds to the "Numbering" column in Tables S11 and S12 where

individual gene abundance and fold change values can be found. Com. (%): community (%) as defined in caption from Table 1.

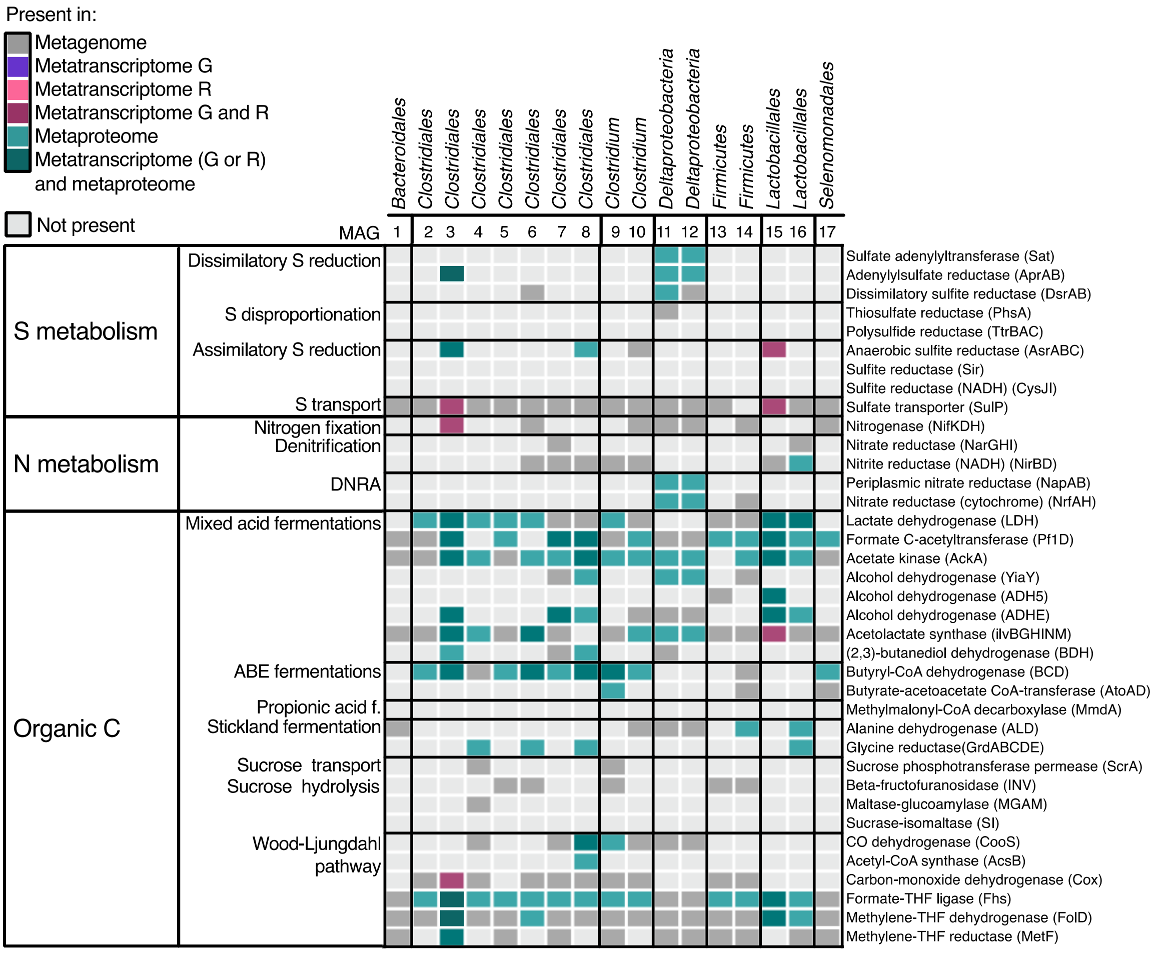

Figure S5. Key enzymes from major metabolic pathways in MAGs from the +As condition EA culture. Vertical bold lines correspond to the grouping of the MAGs with same lineage (Table 1). Pathway abbreviations: dissimilatory nitrate reduction to ammonia (DNRA), organic carbon metabolism (Org. C), propionic acid fermentation (propionic acid f.), and acetone-butanol-ethanol (ABE) fermentation. Refer to Table S16 for individual gene, transcript and protein abundance values and further enzymes.

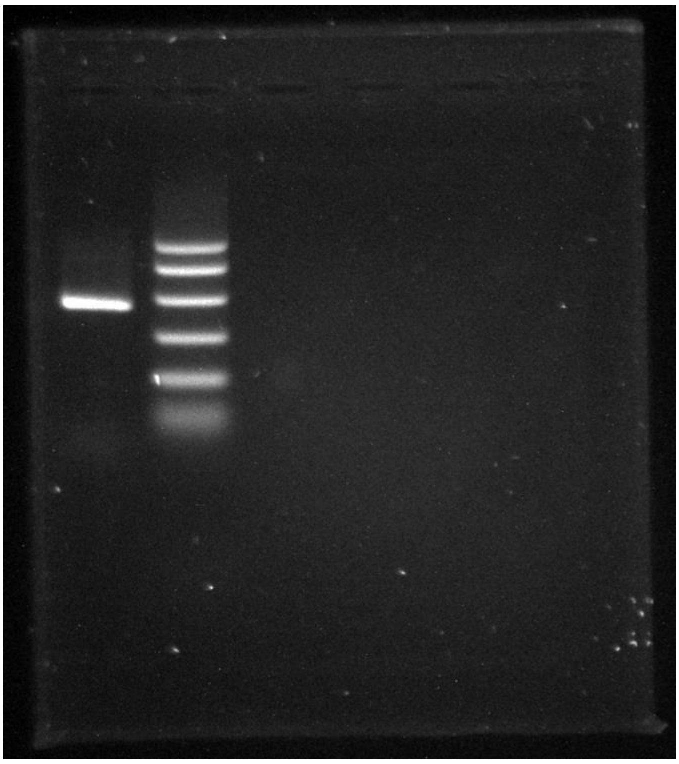

Figure S6. Colony PCR agarose gel from *Paraclostridium* sp. EML. Left lane: PCR product from the amplification of *arsM* (protein id k119_30669_28, Table S11) in the PCR reaction using a *Paraclostridium* sp. EML colony, right lane: ladder corresponding to (from top to bottom) 1000, 750, 500, 300, 150 and 50 bp.

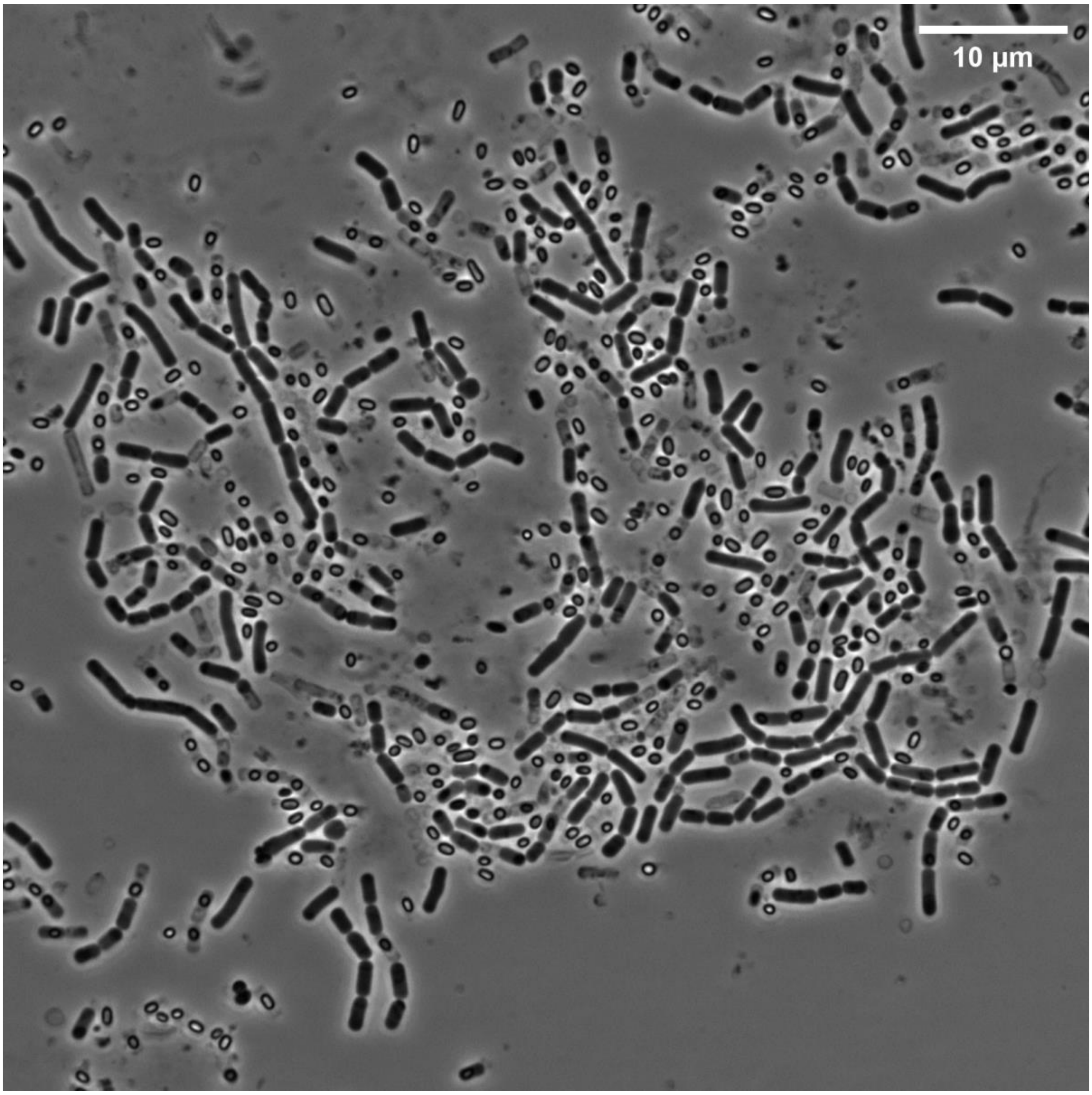

Figure S7. Light microscopy of *Paraclostridium* sp. EML cells, 48-h culture.

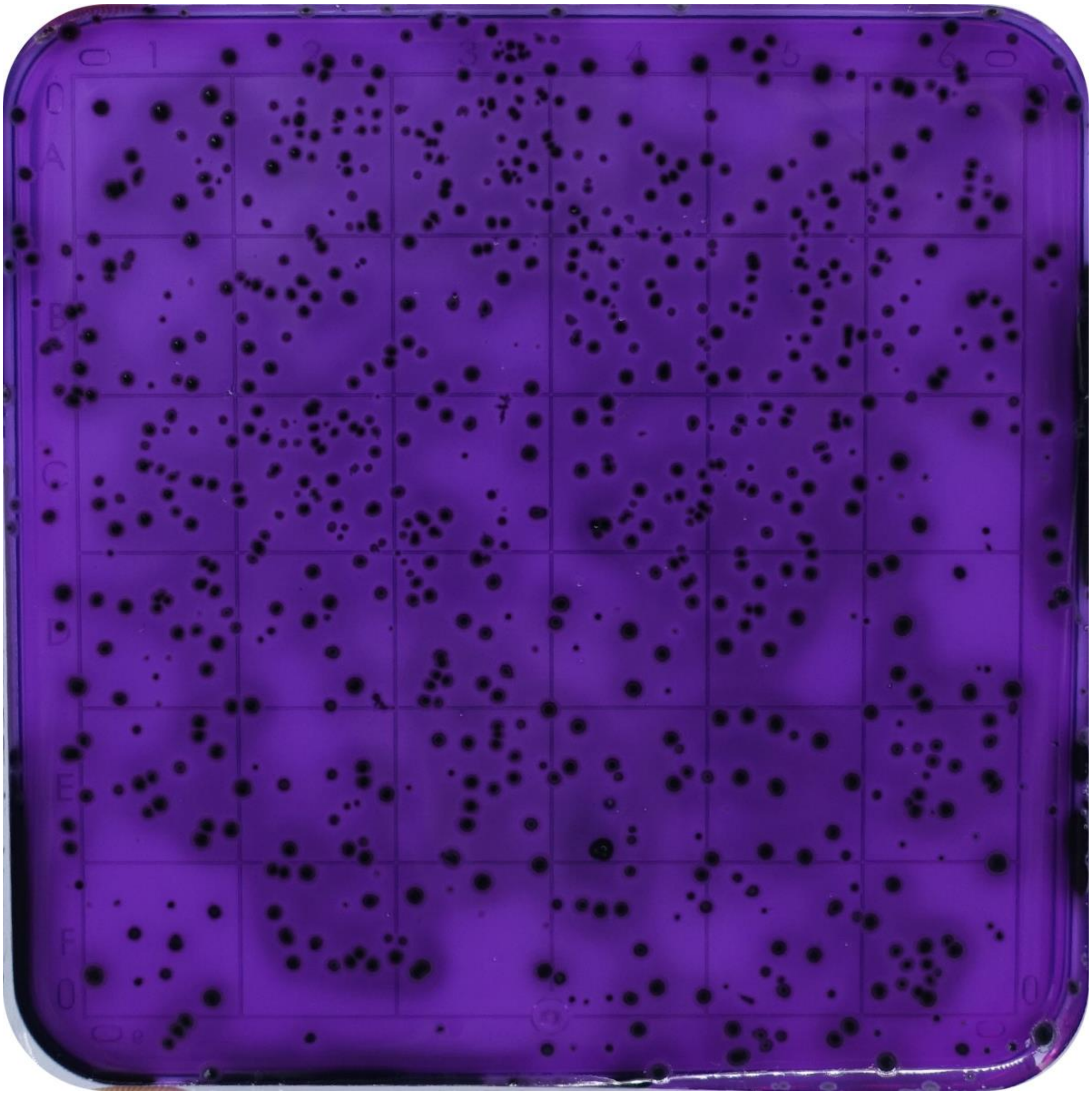

Figure S8. Growth of *Paraclostridium* sp. EML isolate in TSC agar.

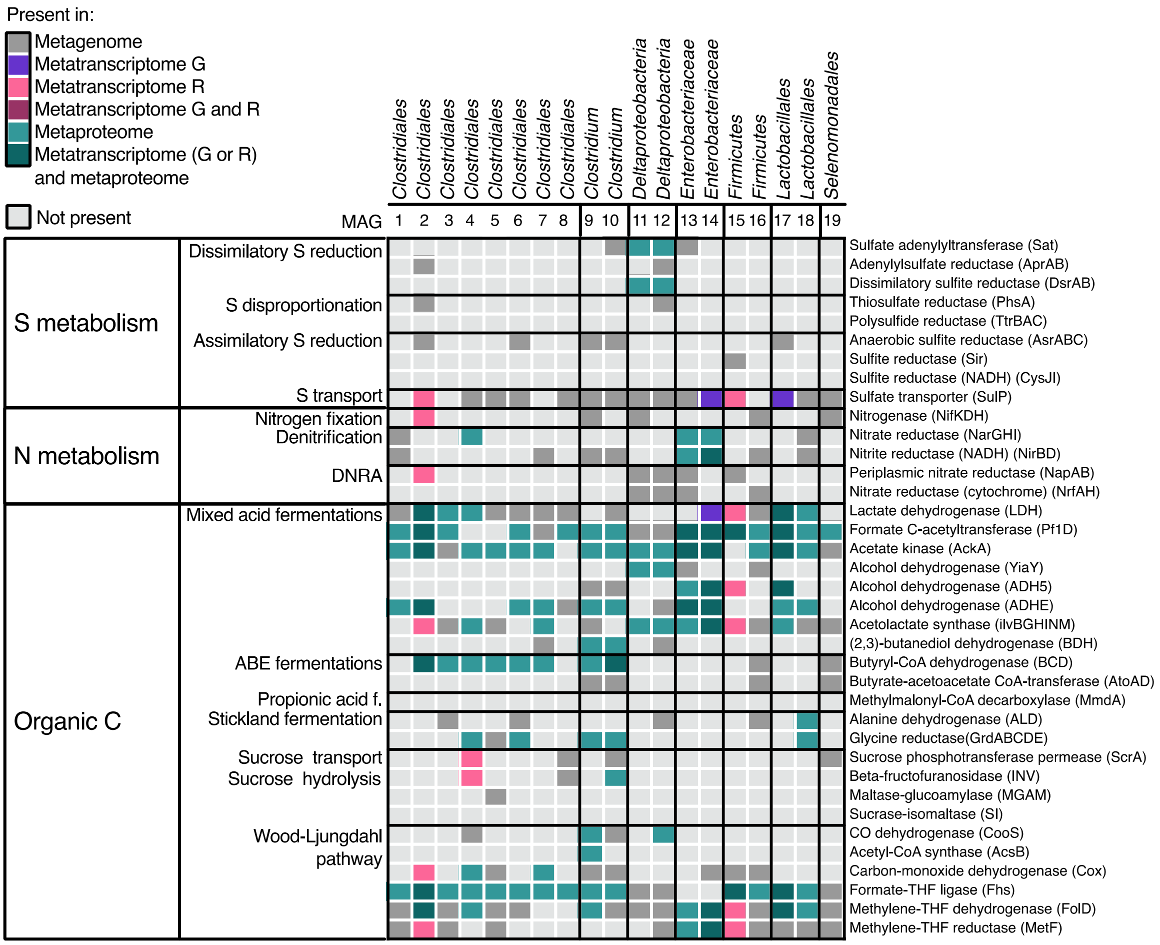

Figure S9. Key enzymes from major metabolic pathways in MAGs from the +As condition TSB culture. Vertical bold lines correspond to the grouping of the MAGs with same lineage (Table 1). Pathway abbreviations: dissimilatory nitrate reduction to ammonia (DNRA), organic carbon metabolism (Org. C), propionic acid fermentation (propionic acid f.), and acetone-butanol-ethanol (ABE) fermentation. Refer to Table S17 for individual gene, transcript and protein abundance values and further enzymes.

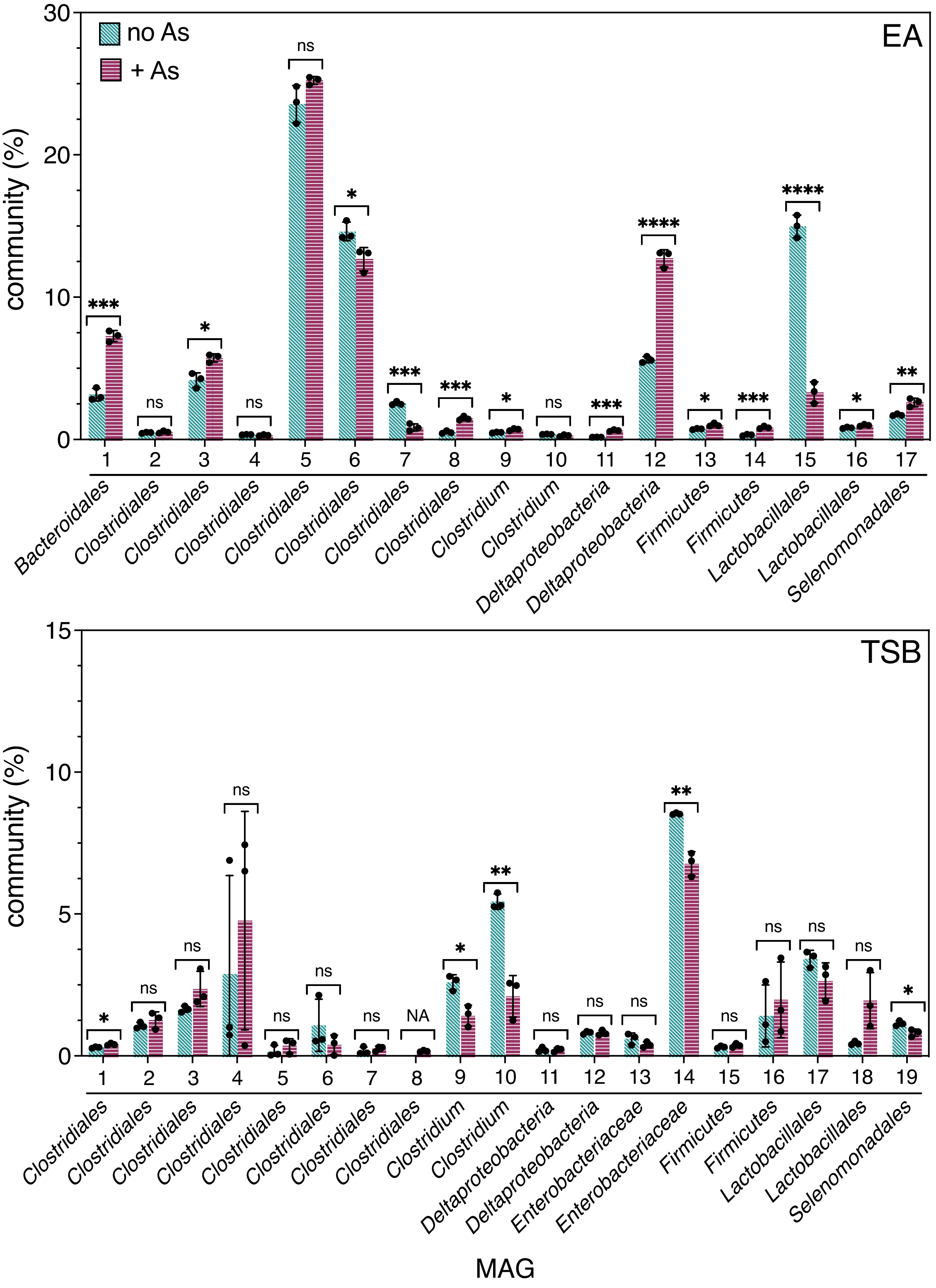

Figure S10. Community (%) of MAGs. Community (%) of MAGs in +As condition and no-As control from EA (top panel) and TSB (low panel) cultures. Statistical differences between +As condition vs. no-As control were identified by unpaired Student t test with *p* value

≤0.05. Symbols: NA: no matching MAG in no-As control was found, one or more asterisks (*): significant difference and ns: no significant difference (*p* value >0.05) (see Table S5 for *p* value symbol summary). Points represent individual values from three biological replicates. Bar heights represent mean and horizontal lines plus, minus one standard deviation.

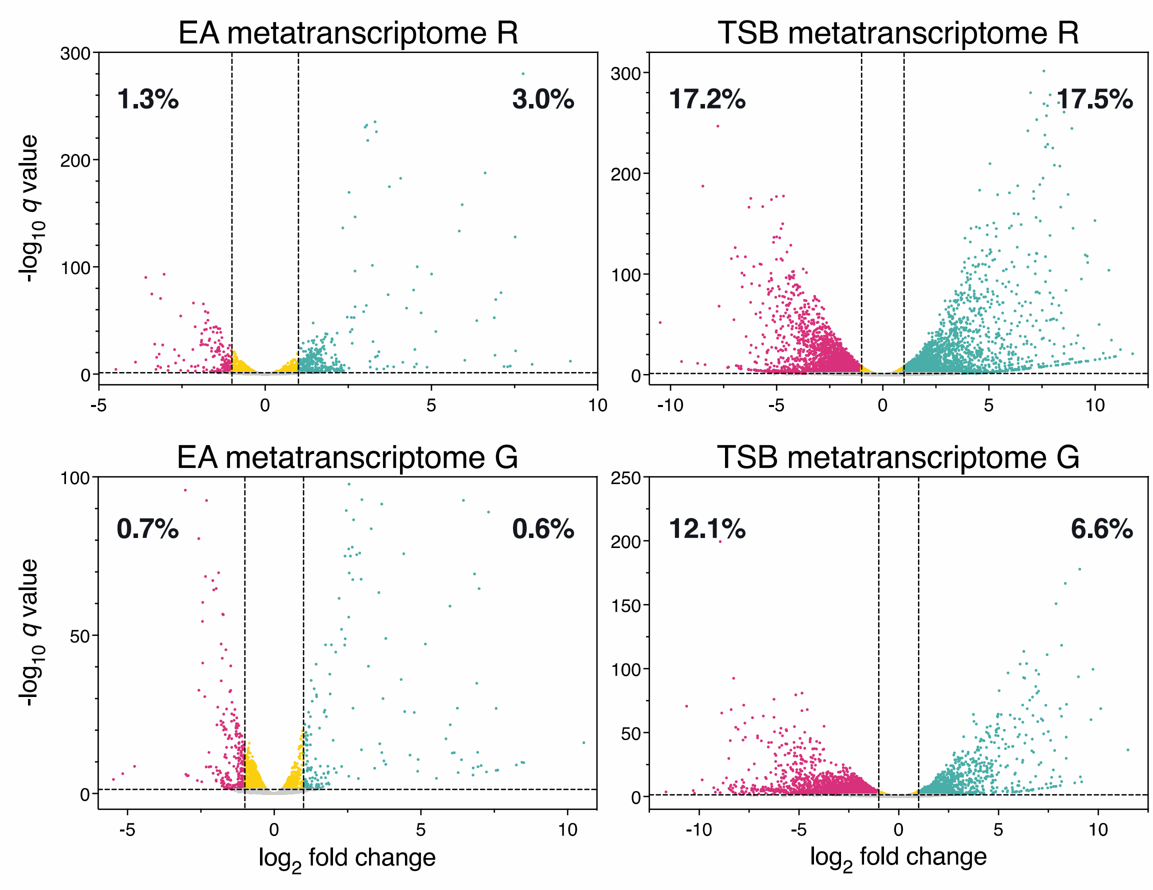

Figure S11. Volcano plots of metatranscriptomes. Dots represent individual genes transcribed in metatranscriptomes R (upper panels) or G (lower panels) from +As condition EA (left panels) and TSB (right panels) cultures. Genes considered statistically differentially transcribed in the +As condition vs. no-As controls, based on the adjusted p value (q value), are represented as magenta (decreased transcription), green (increased transcription) and yellow (-1< $\log_2$ fold changes < 1) dots. Grey dots are genes with non-statistically significant changes in transcription. In bold numbers, the percentage of genes in magenta or green. Individual fold-change values are available in Tables S19 and S20.

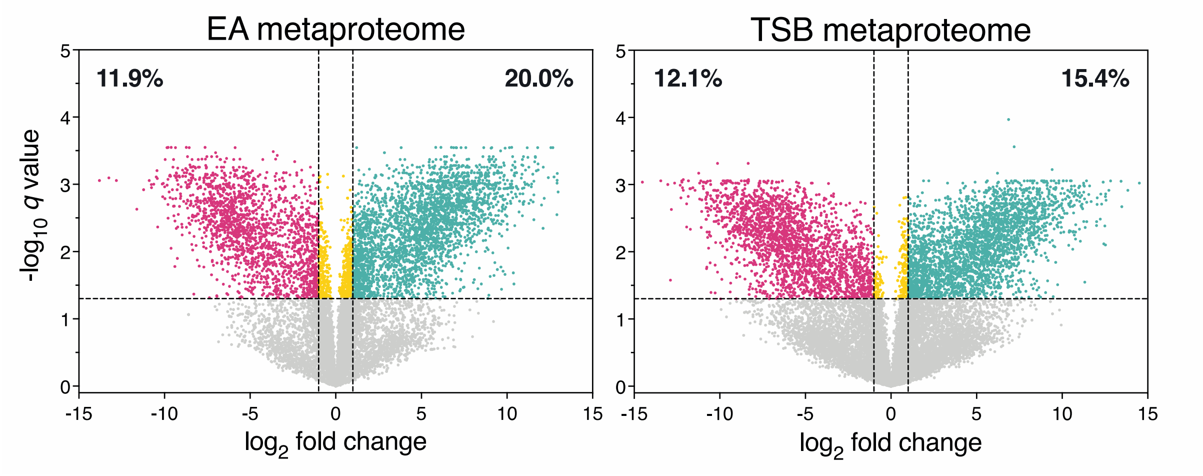

Figure S12. Volcano plots of metaproteomes. Dots represent individual genes expressed in metaproteomes from +As condition EA (left panel) and TSB (right panel) cultures. Genes considered statistically differentially expressed in the +As condition vs. respective no-As controls, based on the adjusted p value (q value), are represented as magenta (decreased expression), green (increased expression) and yellow (-1< $\log_2$ fold changes < 1) dots. Grey dots are genes with non-statistically significant changes in expression. In bold numbers, the percentage of genes in magenta or green. Individual fold-change values are available in Tables S19 and S20.

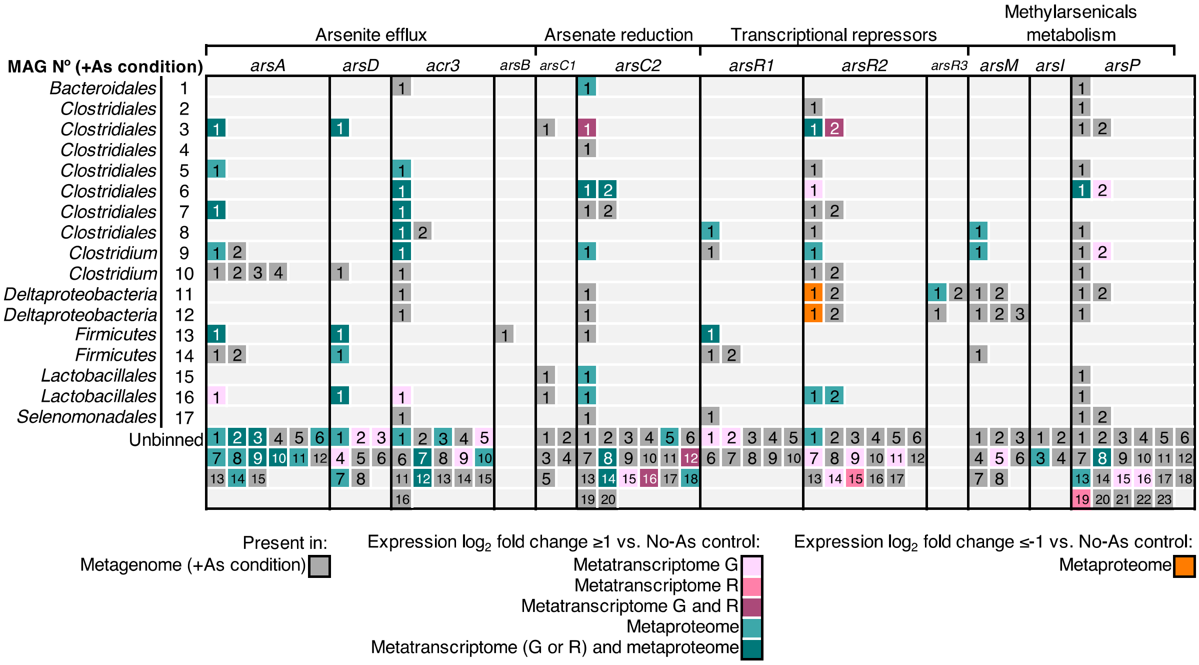

Figure S13. Distribution of *ars* genes encoded in MAGs from the +As condition EA

culture and differentially expressed in metatranscriptomes/metaproteome relative to the no-As EA control. Each numbered box represents an *ars* gene. The number in each box corresponds to the "Numbering" column in Table S11 where individual gene abundance and fold change values can be found.

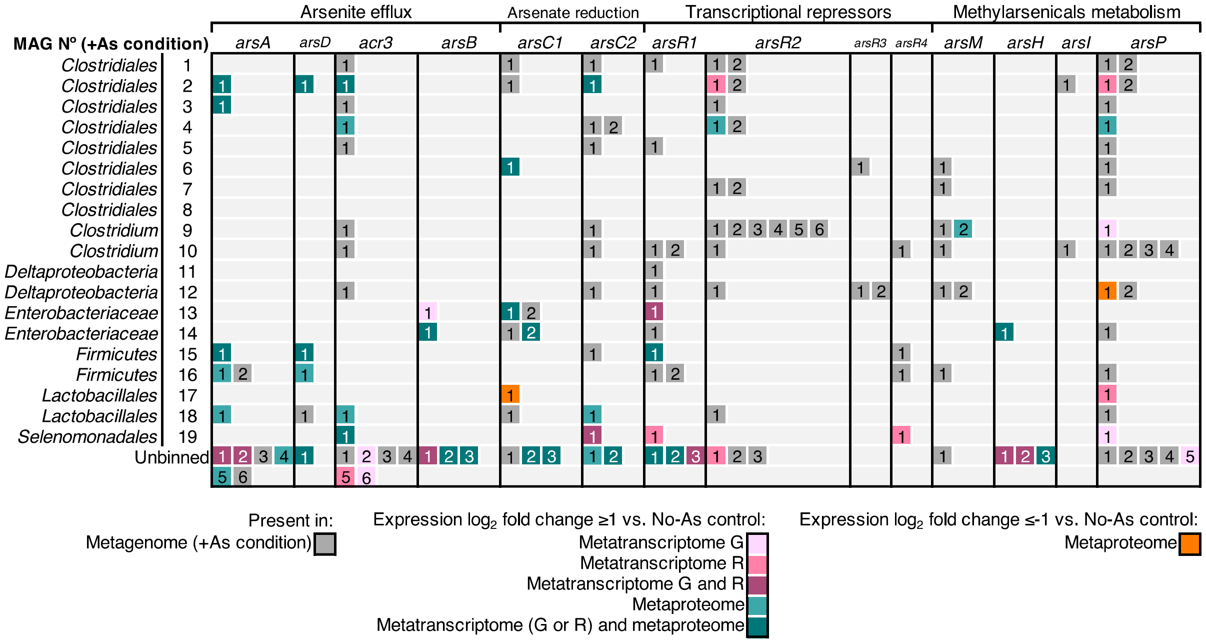

Figure S14. Distribution of *ars* genes encoded in MAGs from the +As condition TSB culture and differentially expressed in metatranscriptomes/metaproteome relative to the no-As TSB control. Each numbered box represents an *ars* gene. The number in each box corresponds to the "Numbering" column in Table S12 where individual gene abundance and fold change values can be found.

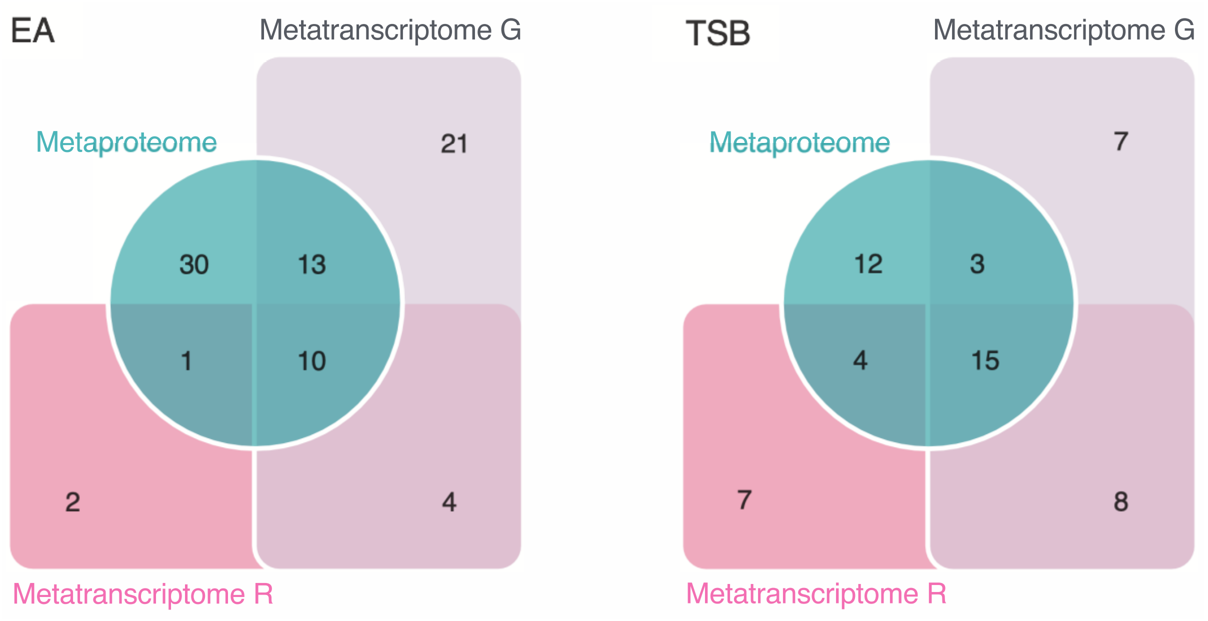

Figure S15. Edwards-Venn diagrams of *ars* genes with increased expression in +As condition EA and TSB relative to no-As control cultures. Number of *ars* genes encoded in metagenomes, with increased expression in metatranscriptomes R and G or/and metaproteomes from +As condition EA culture (left panel) and +As condition TSB (right panel) cultures.

References

1. DeLong EF. Archaea in coastal marine environments. *PNAS* 1992; **89**: 5685–5689.
2. Edwards U, Rogall T, Blöcker H, Emde M, Böttger EC. Isolation and direct complete nucleotide determination of entire genes. Characterization of a gene coding for 16S ribosomal RNA. *Nucleic Acids Res* 1989; **17**: 7843–7853.
3. Viacava K, Meibom KL, Ortega D, Dyer S, Gelb A, Falquet L, *et al*. Variability in arsenic methylation efficiency across aerobic and anaerobic microorganisms. *Environ Sci Technol* 2020; **54**: 14343–14351.
4. Reid MC, Maillard J, Bagnoud A, Falquet L, Le Vo P, Bernier-Latmani R. Arsenic methylation dynamics in a rice paddy soil anaerobic enrichment culture. *Environ Sci Technol* 2017; **51**: 10546–10554.
5. Halkman HBD, Halkman AK. Indicator Organisms. In: Batt C, Patel P (eds). *Encyclopedia of Food Microbiology*, 2nd ed. 2014. Elsevier, pp 358–363.
6. Müller B, Sun L, Schnürer A. First insights into the syntrophic acetate-oxidizing bacteria - a genetic study. *Microbiologyopen* 2013; **2**: 35–53.
7. Huerta-Cepas J, Szklarczyk D, Heller D, Hernández-Plaza A, Forslund SK, Cook H, *et al*. eggNOG 5.0: A hierarchical, functionally and phylogenetically annotated orthology resource based on 5090 organisms and 2502 viruses. *Nucleic Acids Res* 2019; **47**: D309–D314.
8. Chen S-C, Sun G-X, Yan Y, Konstantinidis KT, Zhang S-Y, Deng Y, *et al*. The Great Oxidation Event expanded the genetic repertoire of arsenic metabolism and cycling. *PNAS* 2020; **117**: 10414–10421.
9. Rosen BP, Bhattacharjee H, Zhou T, Walmsley AR. Mechanism of the ArsA ATPase. *Biochim Biophys Acta - Biomembr* 1999; **1461**: 207–215.
10. Johnson LS, Eddy SR, Portugaly E. Hidden Markov model speed heuristic and iterative HMM search procedure. *BMC Bioinformatics* 2010; **11**: 431.
11. Yang Y, Wu S, Lilley RM, Zhang R. The diversity of membrane transporters encoded in bacterial arsenic-resistance operons. *PeerJ* 2015; **3**: e943.
